# Tissue-Dependent Adaptations and Functions of Innate Lymphoid Cells

**DOI:** 10.3389/fimmu.2022.836999

**Published:** 2022-03-10

**Authors:** Julia M. Murphy, Louis Ngai, Arthur Mortha, Sarah Q. Crome

**Affiliations:** ^1^Department of Immunology, University of Toronto, Toronto, ON, Canada; ^2^Toronto General Hospital Research Institute, University Health Network, Toronto, ON, Canada; ^3^Ajmera Transplant Centre, University Health Network, Toronto, ON, Canada

**Keywords:** innate lymphoid cell (ILC), NK cell, tissue-resident immune cells, tissue homeostasis, autoimmunity, inflammation, immune tolerance

## Abstract

Tissue-resident immune cells reside in distinct niches across organs, where they contribute to tissue homeostasis and rapidly respond to perturbations in the local microenvironment. Innate lymphoid cells (ILCs) are a family of innate immune cells that regulate immune and tissue homeostasis. Across anatomical locations throughout the body, ILCs adopt tissue-specific fates, differing from circulating ILC populations. Adaptations of ILCs to microenvironmental changes have been documented in several inflammatory contexts, including obesity, asthma, and inflammatory bowel disease. While our understanding of ILC functions within tissues have predominantly been based on mouse studies, development of advanced single cell platforms to study tissue-resident ILCs in humans and emerging patient-based data is providing new insights into this lymphocyte family. Within this review, we discuss current concepts of ILC fate and function, exploring tissue-specific functions of ILCs and their contribution to health and disease across organ systems.

## Introduction

Innate lymphoid cells (ILCs) orchestrate immune responses to signals such as cytokines, alarmins, neuropeptides and hormones, interacting with hematopoietic and non-hematopoietic cells alike. ILCs lack rearranged antigen receptors and while predominantly tissue-resident, are also observed in circulation and secondary lymphoid tissues where they exhibit distinct spatial and temporal functions (1). Outside of roles in immunity, ILCs have key roles in maintaining tissue homeostasis, promoting tissue repair, and regulating inflammation. *Via* crosstalk with parenchymal cells, ILCs are also involved in processes previously thought to lack immune system influence, such as thermal regulation, neuronal signal transduction, circadian rhythms, and tissue remodeling (2–6). The regulation of both immune functions and tissue-specific processes by ILCs highlights the importance of understanding how they respond and function within tissue niches, and conversely how ILC biology is controlled by the microenvironment in which they reside.

**Figure 1:**
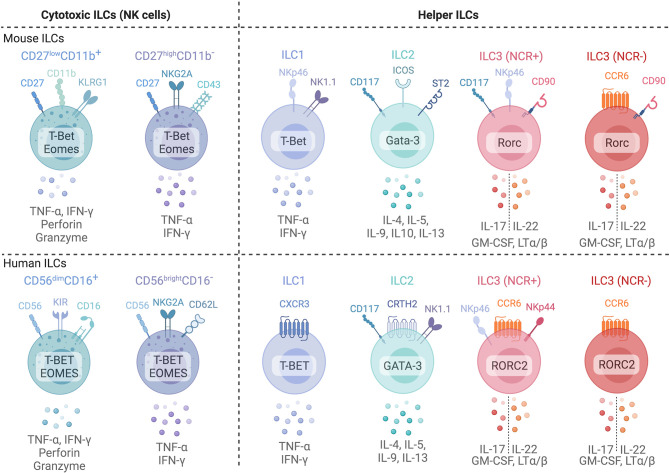
Common phenotypic markers of mice (top) and human (bottom) ILCs and their common cytokine expression profiles. Cytotoxic Natural Killer cells can be subdivided into two major subsets based on surface marker expression in both mice and humans. In mice, NK cells are subdivided into two subsets based on CD27 and CD11b expression: CD27^high^CD11b^-^ immature NK cells and mature CD27^low^CD11b^+^ NK cells. In humans, CD56^bright^CD16^-^ and CD56^dim^CD16^+^ are generally used to identify immature and mature NK cells in blood. However, tissue NK cells often display a CD56^bright^CD16^-^ phenotype. ILC1s, ILC2s, and ILC3s are classified based on surface marker and transcription factor expression profiles that parallel CD4^+^ T helper subsets. ILC3s are further subdivided into natural cytotoxicity receptor (NCR)^+^ and NCR^-^ subsets. Created with Biorender.org.

Development of ILCs in non-lymphoid tissues occurs when circulating ILC progenitors seed tissue niches, and requires the expression of local survival factors including IL-7 and thymic stromal lymphopoietin (TSLP) (7, 8). Differentiated ILCs express signature cytokines and transcription factors that parallel CD4^+^ and CD8^+^ T cells in both humans and mice **(** **Figure 1**) (6, 8), and can be broadly categorized as cytotoxic (NK cells) or non-cytotoxic ‘helper’ ILCs. Human NK cells express TBET and Eomesodermin (EOMES), release IFN-γ and TNF-α and are grouped into CD56^dim^CD16^+^ or CD56^bright^CD16^-^ NK cells. CD56^dim^CD16^+^ NK cells express killer cell immunoglobulin-like receptors (KIRs) and exhibit profound cytotoxic potential (6, 8). CD56^bright^CD16^-^ NK cells lack KIR expression but are superior producers of IFN-γ and TNF-α (9, 10). NK cells discriminate between self and non-self or altered-self and function in anti-viral and anti-tumor immunity similar to CD8^+^ cytotoxic T cells (6, 8). ‘Helper’ ILC (hILCs) are non-cytotoxic and are classified based on function and development into Group 1 (ILC1s), Group 2 (ILC2s), Group 3 (ILC3s) as well as Lymphoid Tissue inducer LTi cells (6). ILC1s produce IFN-γ in a TBET-dependent but EOMES-independent manner (6). ILC2s express GATA-3 and RORα and secrete interleukin (IL)-4, IL-5, IL-9, IL-13 and Amphiregulin (AREG), aiding in anti-parasite immunity or the promotion of allergic responses (6). ILC3s rely on the transcription factor RORC and produce IL-22, IL-17, and GM-CSF (6). ILC3s include subsets which express natural cytotoxicity receptors (NCRs) NKp44 (human) and NKp46 (mouse and human). LTi cells express ILC3-associated transcription factors and cytokines but also express surface Lymphotoxin (sLT) (11). Further, ILCs with immunosuppressive activity have been identified in cancer, intestinal inflammation, allergy, autoimmunity and ischemia reperfusion injury (12–18). These include both NK-like ILCs, IL-10 producing ILC2s (ILC2_10_) and ID3^+^ regulatory ILCs [reviewed in Jegatheeswaran et al. (19)]. Despite growing appreciation of ILCs with regulatory functions, their development and function are poorly characterized, particularly in humans.

**Figure 2:**
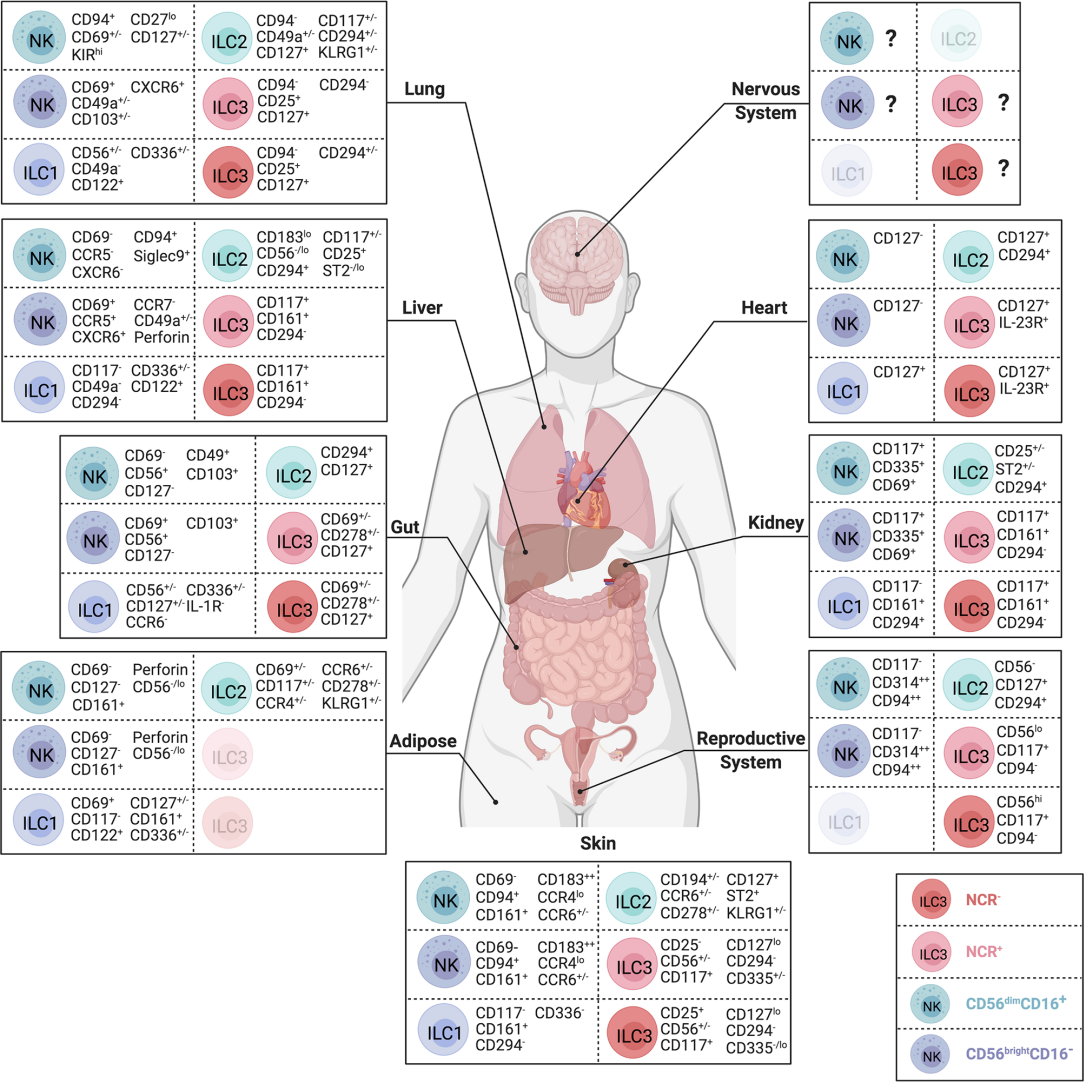
Body-wide distribution and surface phenotypes of human ILCs. Surface marker expression of CD56^dim^ NK cells (teal), CD56^bright^NK cells (dark blue), ILC1s (purple), ILC2s (green), NCR^-^ ILC3s (red) and NCR^+^ ILC3s (pink) in nervous system, lung, heart, liver, kidney, gut, reproductive system, adipose tissue, and skin. ILC subsets that have not yet been identified during steady state visualized in lighter color. Represented selection of ILC markers is based on the consistent use of these markers across multiple independent studies. Created with Biorender.org.

Mouse studies identified central roles for ILCs in regulating tissue homeostasis, repair and remodeling, transforming our understanding of cellular interactions between immune cells and the tissues in which they reside. Across tissue microenvironments, ILCs adapt and acquire distinct phenotypes and functional properties (**Figure 2**). While ILC subsets have important functions within these tissues, dysregulation of ILC numbers and functions is associated with diverse human pathologies including arthritis, diabetes, psoriasis, asthma, and inflammatory bowel disease [reviewed in (20)], highlighting the need to identify how local tissue factors promote or inhibit inflammatory ILC responses. Within this review, we explore NK cell and hILC biology across different tissues in health and disease, highlighting evidence of similarities between human and mouse ILC function where data is available. We summarize current understanding of organ-specific functions of ILCs, focusing on their contributions to tissue homeostasis, host-defense, and inflammatory disease progression across the body.

## ILCs in the Nervous System

While the central nervous system (CNS) is considered an immune-privileged site with minimal immune infiltrate, ILCs have been identified in the CNS of healthy humans and mice, accounting for ~2.5% of leukocytes by sequencing (21–25). CNS-resident NK cells are present in low proportions in the naïve mouse brain and enriched in a IL-2R^+^ CD27^+^ CD62L^high^ subset, suggesting a more mature phenotype compared to infiltrating NK cells (22). CNS ILC2s accumulate with age and reside in the healthy murine meninges, localizing within dural sinuses and surrounding blood vessels (23–25). Interestingly, the transcriptional profile of meningeal ILC2s showed downregulation of genes related to metabolism, signal transduction, and inflammation compared to lung-derived ILC2s, suggesting a tissue-specific quiescent adaptation to the CNS environment (23). Upon spinal cord injury in mice, ILC2s migrate to the injured site independently of IL-33 and upregulate *Calca* (CGRP) and its receptor *Ramp3*, associated with nerve regeneration (23), yet the regenerative activity of ILC2s in the spinal cord remains to be demonstrated experimentally.

### ILCs in Multiple Sclerosis

**Figure 3:**
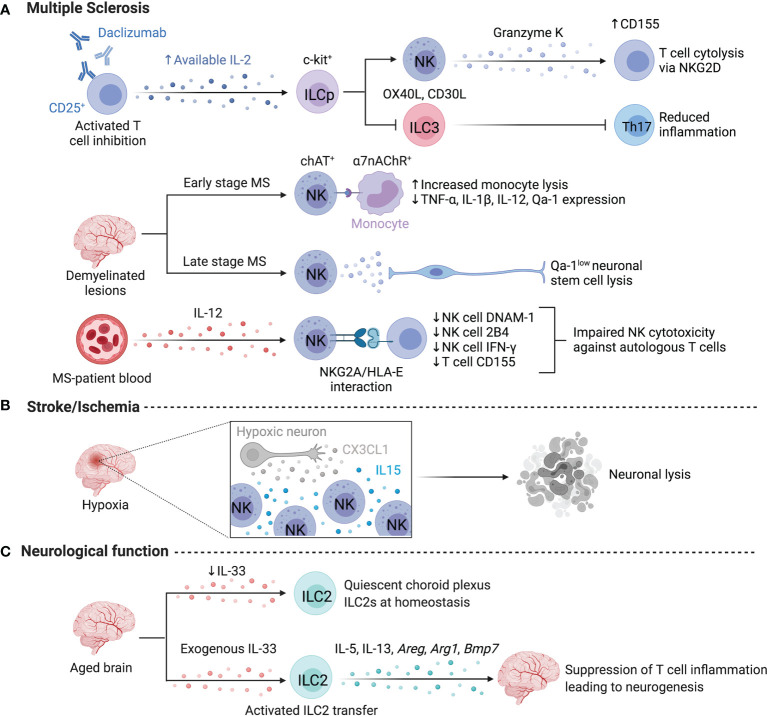
ILCs in the nervous system. Limited information exists on human ILCs in the nervous system at steady state due to challenges in obtaining samples, however, several studies focus on ILC activity in multiple sclerosis or stroke. **(A)** Daclizumab-driven inhibition of T cells resulted in the expansion of NK cells and the elevated lysis of T cells. Daclizumab treatment lowers the abundance of Lin^-^c-kit^+^RORC2^+^ ILCs and dampens Th17-associated inflammation by lowering IL-17 and GM-CSF. ChAT^+^ NK cells preferentially localize to demyelinated lesions in the human brain to dampen monocyte-driven inflammation *via* the a7-nicotinic acetylcholine receptor (a7nAChR), rendering myeloid cells more susceptible to lysis at early stages of MS. Conversely, NK cell-mediated lysis negatively impacts regeneration during later stages of MS by targeting, Qa-1^low^ neuronal stem cells. NK cell activation may be impaired by NKG2A/HLA-E interactions with autologous CD4^+^ T cells. **(B)** After a stroke, CX3CL1 from hypoxic neurons recruits NK cells, while local IL-15 levels facilitate NK cell enrichment and promote high NKG2D expression and neuronal lysis. **(C)** ILC2s impact neurological functions in murine brain and are supported by exogenous IL-33 to suppress T cell inflammation and enhance neurogenesis. Created with Biorender.org.

Multiple sclerosis (MS) is a demyelinating and neurodegenerative autoimmune disease that is one of the most common neurological disabilities in young adults (26). NK cells mediate several treatment-related effects in MS patients (**Figure 3A**). For example, Daclizumab targets the high affinity IL-2 receptor (CD25), inhibiting activated T cells and resulting in greater availability of IL-2, which expands CD56^bright^ NK cells expressing high levels of the medium-affinity IL-2 receptor chain (CD122) (18, 27). This expansion of CD56^bright^ NK cells or elevated baseline expression of CD122 in patients correlated with lower inflammation and fewer inflammatory lesions (18, 28). While T cells are only modestly depleted by Daclizumab directly, induction of T cell apoptosis by CD56^bright^ NK cells is supported by findings of Granzyme K^+^ NK cell co-localization with T cells in active MS lesions (18, 29, 30). Takahashi et al. further found that during the remission phase of MS, CD95 expression increased on NK cells alongside decreased response of memory T cells, suggesting that CD95^+^ NK cells regulate autoimmune memory T cell responses during remission (31). In autologous hematopoietic stem cell transplantation, another MS treatment modality, NK cells reconstitute faster than CD4^+^ T cells and regulate disease-promoting Th17 cells *via* NKG2D-mediated cytotoxicity, preventing lesion formation and relapse (32).

A higher ratio of CD56^bright^ to CD56^dim^ NK cells is observed in the cerebrospinal fluid of patients with MS compared to those with other inflammatory and non-inflammatory neurological diseases, suggesting an MS-specific alteration in resident NK cells with controversial effects on the abundance of NK cells in circulation (30, 33, 34). Despite conflicting findings regarding abundance, circulating CD56^bright^ NK cells from MS patients have reduced IFN-γ production in response to IL-12 and an impaired ability to regulate autologous CD4^+^ T cells compared to healthy controls (33, 35). This impaired regulatory capacity was due to HLA-E upregulation on autologous T cells engaging the inhibitory receptor NKG2A on NK cells (35). Further, DNAM-1 and 2B4 were reduced on NK cells alongside reduced expression of the DNAM-1 ligand CD155 on CD4^+^ T cells, while Daclizumab treatment induced CD155 upregulation on T cells to partially rescue the impaired ability of NK cells to regulate autologous T cells (30). A genome-wide association study of MS patients demonstrated lower expression of *TBX21* and *EOMES* in NK cells, supporting that impairment of NK cells may be a driver of MS (36).

Using the experimental autoimmune encephalomyelitis (EAE) model of MS, Hao et al. demonstrated the importance of CX3CR1-mediated recruitment in generating disease-ameliorating CNS-resident mouse NK cells (37). Transmigration of NK cells into the CNS partially depends on VLA-4 binding to endothelial VCAM-1, as antibody blockade of VLA-4 reduces NK cell recruitment by 40-70% (30, 38). Absence from or blocked transmigration results in excessive proliferation of myelin-reactive CD4+ T helper 17 (Th17) cells, indicating that NK cells must be within the CNS to limit myelin-specific T cell activity and disease progression (37). Mouse NK cell-mediated disease amelioration required an NCR- and perforin-dependent lysis of microglia to abrogate Th17 expansion (37). The tight proximity of microglia and NK cells requires reciprocal chemoattraction through secretion of MIP-1α and MCP-1 by NK cells and microglia, respectively (37, 39). Additionally, NK cells dampen EAE pathogenesis by directly modulating infiltrating CCR2^+^Ly6C^hi^ monocytes in an acetylcholine-dependent fashion. Adoptive-transfer of choline acetyltransferase (ChAT)-expressing NK cells into the CNS of *Cx3cr1*^-/-^ mice reduced the abundance of infiltrating monocytes (40). ChAT^+^ NK cells dampened TNF-α, IL-1β, IL-12 and Qa-1 expression by monocytes through engaging the α7-nicotinic acetylcholine receptor, rendering myeloid cells more susceptible to lysis (40). ChAT^+^ NK cells preferentially localize to active demyelinated lesions in the human brain, suggesting this mechanism of microglial regulation may translate to human MS as well (40). While dampening myeloid and T cell activity reduces disease severity, murine NK cells negatively impact regeneration through lysis of Qa-1^low^ neuronal stem cells in the sub-ventricular zone, altering neuronal repair and impairing recovery in later disease (41). Of note, NK cell activation *via* NKG2D triggered motor neuron destruction in models of amyotrophic lateral sclerosis, suggesting pathological NK cell-mediated lysis of neurons is not specific to MS/EAE (42).

Other ILCs have been identified in MS too, although inconsistent phenotyping has hindered identification of these ILCs. A sizable fraction of CD3^-^ IL-17^+^ RORγt^+^ cells associate with newly formed meningeal lymphoid follicles of MS patients, suggestive of ILC3 involvement (43). In mice, CD3^-^RORγt^+^ populations in the cerebellum after EAE induction were predominantly CD4^-^, consistent with ILC3 identity (44). Hatfield et al. reported both NCR^+^ and NCR^-^ ILC3s and CD4^+^ CD3^-^ LTi-like ILC3s within the meninges of healthy mice which proliferated and accumulated downstream of c-kit signaling during EAE induction (45). Meningeal ILC3s produce IL-17 and GM-CSF, and express co-stimulatory molecules OX40L and CD30L. They accumulated near Th17 cells and antigen presenting cells (APCs) and facilitated T cell activation and entry into the brain parenchyma in a T-bet-dependent fashion, highlighting a role for ILC3s in establishing a microenvironment that sustains Th17 responses in EAE (45, 46).

Helper ILCs (hILCs) are also affected by Daclizumab treatment and appear to play a sex-biased role in MS/EAE. Untreated MS patients presenting with elevated white blood cell counts displayed higher levels of RORγt^+^ ILCs in their cerebrospinal fluid (47). Daclizumab treatment lowered CXCL13 levels and the abundance of Lin^-^c-kit^+^RORγt^+^ ILCs, suggesting that ILC3 inhibition may be another beneficial effect of Daclizumab treatment (48). *In vitro* differentiation of c-kit^+^ ILC precursors and CD34^+^ hematopoietic progenitor cells under high IL-2 conditions favored the development of CD56^bright^ NK cells and restrained ILC3 differentiation, implying that greater *in vivo* IL-2 availability affects the development of ILCs by altering subset composition (48). MS has a higher prevalence in females and is correlated with reduced accumulation of ILC2s in EAE models (49). Interestingly, male mice that have reduced c-kit signaling (Kit^W/Wv^) failed to accumulate ILC2s and adopted a female disease phenotype suggesting a sex-dependent role for ILC2s in protection from EAE pathogenesis (49). *Il33* expression is only upregulated in male mice after myelin peptide immunization, and IL-33 administration in female mice expands ILC2s and provides protection from EAE, while anti-IL-33 treatment abrogates protection in male mice, further supporting sex effects on ILC2 function, dependent on differential IL-33 availability (50).

### ILCs in Cerebral Ischemia (Stroke)

After a stroke, human peripheral blood NK cells are reduced early (< 72h) and the degree of reduction as well as expression of activation markers positively correlates with infarct volume (51, 52). Within 12 hours of intracerebral hemorrhage, CD69^+^Perforin^+^ NK cells become the dominant immune cell type in perihematomal regions (21). 24h following a stroke, CD69^+^NKp46^+^ cell numbers peaked in the brain and remained elevated (52). In mice, the accumulation of NK cells during the acute phase of stroke is mediated by the release of CX3CL1 by hypoxic neurons (53). Recruited NK cells accumulate in an IL-15-rich environment, adopt an activated phenotype, and mediate neuronal lysis through missing-self activation (**Figure 3B**) (53). Ischemia-reperfusion injury (IRI) induces IL-15 production by neurons, astrocytes and microglia, blockade of which reduced IFN-γ^+^ NK cells in the murine brain (54). Liu et al. reported that cholinergic signaling in the brain and catecholaminergic signaling in the periphery suppressed NK cell function after cerebral ischemia, contributing to post-stroke susceptibility to infection (52). While adrenergic activation suppressed NK cell abundance and function in the periphery, cholinergic signaling reduces *Runx3* expression in CNS NK cells, leading to a decline in NK cell responsiveness and demonstrating the involvement of distinct neural pathways in regulating the spatial activation of NK cells in mice and humans (52). In humans, the microRNA (miRNA) profile of peripheral NK cells is altered after stroke and inhibition of miRNA-451a and miRNA-122-5p partially restored CD69 and NKG2D expression, suggesting that targeting miRNAs may alleviate immunosuppression observed after a stroke (51). Although data supporting a role for helper ILCs in response to stroke is scarce, early after an acute cerebral infarction circulating ILC1s increased and ILC2s decreased, correlating to serum ox-LDL levels, suggesting lipid-mediated regulation of ILC1 and ILC2 abundance (55).

### ILCs in Neurological Function

Murine studies support a role for ILCs in regulating neurological function. Depletion of NK cells using anti-NK1.1 improved cognitive function, enhanced neurogenesis, and reduced microglial inflammation but did not affect β-amyloid concentration in a mouse model of Alzheimer’s disease (56). NK cells exhibited altered expression profiles in the disease model, with higher expression of *Icam1*, *Ctsb*, *Ctsc*, *Ccl3* and *Ccl4* (56). Following NK cell depletion, microglia exhibited a return to homeostatic morphology, reduced proliferation, and reduced expression of pro-inflammatory mediators including *Il18*, *Il1a*, *Il1b* and *Tnf*, suggesting that NK cells and type I immunity contribute to cognitive decline by promoting microglial inflammation (56). In line with these findings, choroid plexus ILC2s accumulated and displayed a quiescent state in the aged brain, which was reversed with IL-33 stimulation (57). In comparison to meningeal ILC2s, choroid plexus ILC2s were resistant to senescence and exhibited higher expression of *Arg1* and genes associated with glycolysis that may underlie their enhanced proliferative and cytokine-producing capacity, and suggest niche-specific functionality (57). Intriguingly, activation of ILC2s in aged mice or transfer of activated ILC2s to the aged brain increased cognitive function, potentially through IL-5-mediated suppression of T cell inflammation leading to enhanced neurogenesis (**Figure 3C**) (57). After traumatic brain injury, ILCs are increased in frequency in human meninges and cerebrospinal fluid, and treatment with AMPK-activating metformin in a murine model specifically enhanced IL-10-producing ILC2s and improved neurological outcomes (58). Together, this suggests that ILC2s support neurological function and resolution of inflammation while NK cells exacerbate cognitive decline.

### ILCs in Peripheral Nervous System

Nervous system signaling in the periphery is also impacted by ILC activity. Specialized pro-resolving mediators (SPMs) such as PCTR1 are important for resolving inflammation and promoting tissue repair (59). Acetylcholine promotes the enzymatic activity of ILC3-derived 15-LOX-1, the initiating enzyme in PCTR1 biosynthesis (60). Production of SPMs is regulated by the vagus nerve, and loss of vagus nerve signaling reduced peritoneal ILC3s in mice resulting in poor resolution of *Escherichia coli* infection (60). The circuit between ILC3s, SPMs, and macrophages is key for resolving infection and inflammation in the peritoneum (60).

## ILCs in the Lung

NK cells account for 10-20% of all lymphocytes in human and murine lungs (61–63). Lung NK cells are marked by higher CD57 and KIR expression, and lower CD27, indicative of a mature phenotype (64). Despite their high KIR expression, human lung CD56^dim^CD16^+^ NK cells are hypofunctional and some CD56^bright^ subsets are characterized by the expression of markers associated with tissue-residency (e.g., *CD69*, *ITGA1* (CD49a), *ITGAE* (CD103), and *CXCR6*) (61, 64). A review by Hervier et al. nicely summarizes the development and function of NK cell subsets in the human lung (65). In addition to NK cells, all other helper ILC subsets have been observed in human lung, albeit with conflicting reports on the relative abundance of ILC1s, ILC2s and ILC3s that may reflect small sample sizes, sampling location, or inter-donor heterogeneity (66, 67).

**Figure 4:**
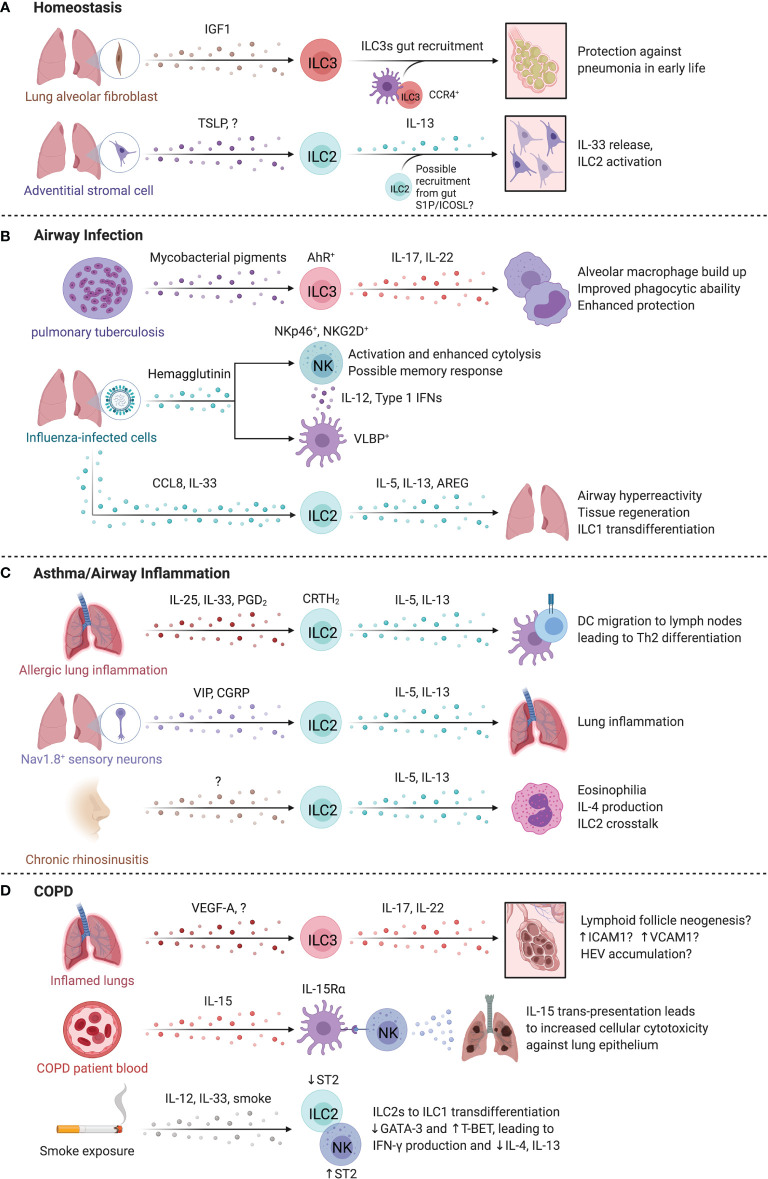
ILCs in the lung. At homeostasis **(A)**, IL-22 producing ILC3s are required for protection against pneumonia and require commensal gut bacteria for their recruitment to the lungs. Once in the lung, ILC3s are sustained locally through insulin-like growth factor 1 (IGF1) from alveolar fibroblasts. In the steady state, Adventitial stromal cell-derived TSLP promotes IL-13 production by ILC2s that drives stromal cells to produce IL-33. **(B)** During pulmonary tuberculosis (PTB), ILC3 accumulation in the lung is regulated by pathogen-derived AhR-ligands that in turn promote phagocyte function, formation of tertiary lymphoid structures and enhanced protection. Similarly, infection by influenza virus triggers NKp46-dependent activation of NK cells resulting in IL-12 and type 1 interferon secretion by DCs that promote NK cell activation. The release of CCL8 and IL-33 during respiratory viral infection facilitated ILC2 activation and AREG-dependent epithelial repair. **(C)** In allergic lung inflammation, IL-13 from ILC2s induced Th2 cell differentiation by promoting migration of activated DCs to the draining lymph nodes. In mice, administration of the CRTH2 ligand, prostaglandin D2, promotes ILC2 accumulation. Nociceptor Nav1.8+ sensory neurons activated lung ILC2s through vasoactive intestinal peptide (VIP), while pulmonary neuroendocrine cells produced calcitonin gene related-peptide (CGRP) collectively promoting allergic inflammation in the murine lung. Nasal polyps accumulate ILC2s in chronic rhinosinusitis, which supports eosinophils and promotes chronic airway inflammation. **(D)** IL-17A^+^IL-22^+^ ILCs and NCR^-^ ILC3s are increased in COPD. The lungs of COPD patients and smokers contain Neuropilin 1 (NRP1)-expressing ILC3s surrounding high endothelial venules. NK cells may contribute to COPD, with higher CD57 expression, IL-15-dependent activation, and greater cytotoxicity against lung epithelial cells. Smoke exposure may lead to a sustained loss of ST2 expression on ILC2s, reducing their responsiveness to IL-33 while paradoxically promoting ST2 expression on NK cells that supports a type 1 response. Created with Biorender.org.

Recruitment as well as local proliferation of ILC precursors in the lung during development shape the pool of tissue-resident ILC subsets (**Figure 4A**). Oherle et al. identified that murine pulmonary ILC3s develop from a local precursor pool sustained by insulin-like growth factor 1 provided by alveolar fibroblasts (68). Early-life seeding of ILC3s was protective against pneumonia in a CCR4-dependent fashion, driven by a gut commensal microbiota – dendritic cell (DC) axis (69). Similar interactions between adventitial stromal cells and mouse ILC2s were reported to sustain and regulate ILC2s homeostasis and function (24, 70). Adventitial stromal cells release TSLP, promoting basal IL-13 release by ILC2s, which in turn activates adventitial stromal cells to produce IL-33 in a homeostatic circuit (24). Interestingly, ILC2s localize around the peribronchial and perivascular adventitial cuff regions independent of microbial signals, IL-25, IL-33 or TSLP, indicating that additional unknown signals regulate pulmonary ILC2 development and recruitment (24, 70, 71). Whether pulmonary ILC2s in mice and humans originate from other tissues at steady state remains unclear, however mouse intestinal ILC2s were demonstrated to traffic to the lungs in an S1P-dependent manner after intraperitoneal IL-25 administration or helminth infection, demonstrating coordination between tissue sites to resolve multi-organ infections (72). Additional niche-signals may be delivered through the ICOS : ICOSL axis that has been demonstrated to sustain the pool of pulmonary ILC2s by elevating anti-apoptotic genes and IL-2 responsiveness (73). Intriguingly, ILC2s express both ICOS and ICOS-L, suggesting that both self-sustaining and helper cell-dependent interactions promote ILC2 homeostasis (73).

### ILCs in Airway Infections

Airborne pathogens are a constant challenge within the lung, and ILCs have a key role in anti-bacterial and anti-viral host defense (**Figure 4B**). Helper ILCs accumulate in the lungs of patients with pulmonary tuberculosis (PTB), while circulating ILCs are reduced (74), suggesting trafficking of ILCs to the lung. ILC3s are critical for host defense in PTB, as specific deletion of ILC3s (Ahr^fl/fl^Rorγt^Cre^) increased mycobacterial burden, and impaired the accumulation of alveolar macrophages and formation of protective lymphoid follicles in granulomas (74). Mycobacterial pigments serve as ligands for Aryl hydrocarbon receptor (AhR), a key transcription factor for ILC3 development and function, suggesting an alternative mechanism of ILC3 activation in tuberculosis infection (75). In addition, ILC3s recruited to murine lungs produced IL-17A and IL-22 to enhance protection and support phagocytic functions of inflammatory monocytes to mediate clearance of bacterial infections (76, 77).

NK cells are critical in controlling viral infections in the lung. Indeed, influenza infection is lethal in *Ncr1^-/-^* mice (78). However, adoptive transfer and antibody-depletion experiments showed that NK cells exacerbated influenza morbidity and mortality in a manner dependent on virus titer (79). Differences in mouse genetic backgrounds, influenza strains, and infectious dosage complicate the interpretation and translation of these findings. In humans, viral hemagglutinin on infected cells triggered NKp46-dependent activation of NK cells, and upregulation of the NKG2D ligand ULBP on infected DCs and elevated secretion of IL-12 and type 1 interferon facilitated NK cell activation and cytolysis in response to influenza (80, 81). In a human lung tissue explant model, CD56^bright^CD49a^+^ NK cells robustly responded to influenza A infection, hinting at an NK cell subset-specific memory response (82). Dou et al. found that seasonal influenza vaccination induced a short-term (6 month) memory response in NK cells, correlating with downregulation of surface NKp46 and a concomitant increase in intracellular NKp46 expression (83). This memory response to re-challenge was not strain-specific, suggesting broader protection to influenza after seasonal strain-specific vaccination (83). While the role of ILC1s separate from NK cells is less clear, murine ILC1s promote antiviral defense and DC maturation, potentially through the glucocorticoid-induced TNFR-related protein (GITR):GITR-L axis (84). GITR upregulation on ILC1s resulted in stronger IFN-γ and TNF-α responses to influenza A, supporting host defense against alveolar viral infections (84).

ILC2s have conflicting roles in influenza infection response, promoting airway hyperreactivity in an IL-13-dependent manner while supporting epithelial cell integrity and tissue repair *via* the secretion of AREG following viral infections (85, 86). In response to CCL8, IL-33-activated ILC2s produce more IL-5 and IL-13, and exhibit ameboid-like movements to traffic to peribronchial and perivascular sites in mice, particularly at locations of increased collagen-I deposition (71). Human ILC2s also exhibited a chemotactic response to CCL8, suggesting shared lung recruitment responses across species (71). Infections with respiratory syncytial virus (RSV) leads to a viral titer-independent increase in respiratory disease severity in young infants driven by elevated ILC2 cytokine release (87, 88). Interestingly, patients older than 3 months had fewer ILC2s in their lungs, greater IFN-γ levels and experienced less severe disease, suggesting that the immunological changes occurring with age and development confer protection to RSV infections by balancing type 1 and type 2 immunity (88). The plasticity of ILC2s may also play a role in promoting type 1 immunity to viral infections. Silver et al. found that adoptively transferred murine ILC2s trans-differentiate into ILC1s near IL-12- and IL-18-expressing myeloid cells during influenza A infection (89). Overall, this suggests that age and plasticity shape ILC2 responses to viral infections.

### ILCs in Asthma and Allergic Airway Inflammation

Asthma is a chronic inflammatory disease of the airways marked by elevated type 2 inflammation (90, 91). ILC2 activity is implicated in airway inflammatory diseases (**Figure 4C**). ILC2-derived IL-13 is critical for inducing Th2 cell differentiation in response to allergic lung inflammation by promoting the migration of activated DCs to the draining lymph nodes, supporting the development of allergic adaptive immune responses (92). Circulating ILC2s from asthmatic patients produced more IL-5 and IL-13 in response to IL-25 and IL-33 stimulation relative to controls, and administration of prostaglandin D_2_, the ligand for CRTh2, promoted ILC2 accumulation in murine lungs (93, 94). A single nucleotide polymorphism resulting in elevated CRTh2 expression positively associates with asthma development in humans, although whether this corresponds directly to increased ILC2 presence is unknown (95). Interestingly, the prevalence of asthma is lower in adult males versus females, indicating sex-specific differences in type 2 immunity (96). Several animal studies recapitulated these sex-dependent changes in abundance, phenotype, and responsiveness of ILC2s and implicated the role of sex-hormones in facilitating sex-specific responses to alveolar diseases (96–100). For example, androgen-receptor signaling negatively regulated ILC2 cytokine secretion and differentiation and reduced IL-33-dependent lung inflammation in male mice (97, 100, 101).

Strikingly, neuronal and neuroendocrine-driven stimulation of ILC2s promotes allergic lung inflammation (102, 103). IL-5-stimulated nociceptor Nav1.8^+^ sensory neurons activated ILC2s through vasoactive intestinal peptide (VIP), while pulmonary neuroendocrine cells trigger ILC2s through the calcitonin gene-related peptide (CGRP) to promote allergic inflammation in the murine lung (102, 103). CGRP-secreting pulmonary neuroendocrine cells were increased in asthmatic patients suggesting that this mechanism could also support ILC2-mediated allergic inflammation in humans, inspiring several pathways of therapeutic interventions (103). Constitutive activation of ILC2s may lead to long-lasting alterations in the lung as found in other pulmonary diseases. For example, ILC2s are enriched in nasal polyps of chronic rhinosinusitis patients along with elevated *IL5* and *IL13* transcripts, suggesting an ILC2-dependent contribution to the disease-associated eosinophilia and chronic airway inflammation (104, 105). Polyp tissues identified with eosinophilia revealed a co-localization of ILC2s and eosinophils, indicating a possible cross-talk between IL-5-producing ILC2s and IL-4-producing eosinophils to support reciprocal activation and survival (106).

Complicating our understanding of ILC2s in allergic responses are recent findings from Golebsky and colleagues that ILC2_10_s are reduced in abundance in allergic individuals relative to non-allergic controls, while sublingual immunotherapy for grass pollen allergy restores this IL-10-producing subset which may confer protection and restoration of epithelial barrier integrity (16). Interestingly, murine lung ICOS^+^ST2^+^ ILC2s exhibit memory in response to allergen challenge dependent on ICOS and IL-33, marked by transcriptional and epigenetic programs involving the scaffold protein *Four And A Half LIM Domains 2* (FHL2) (107). Further, adoptive transfer of FHL2^+^CRTh2^+^ human ILC2s induced airway hyperreactivity in mice and were partially steroid resistant, suggesting memory ILC2s may be relevant to steroid-resistant asthma (107).

Similar to ILC2s, ILC3s have been linked to asthma pathology. IL-17 levels and IL-17^+^ ILC3s were elevated in bronchial alveolar lavage fluid of asthmatic patients, especially in patients with severe disease (108, 109). An ILC3 gene signature was upregulated in nasal brushings of adult-onset severe asthma patients, while bronchial brushings revealed elevated type 2 related gene profiles, supporting the idea of an anatomic preference of distinct ILC responses that may selectively contribute to site-specific characteristics of disease (110).

ILCs also contribute to chronic pulmonary inflammation through regulation of adaptive immune cells. CD40L expression by human and murine T helper cells induces an IgE response by B cells, contributing to airway hyper-responsiveness (111, 112). CD40L expression on T cells is induced by cAMP only in the presence of CD56^+^CD16^+^ NK cells through a contact-dependent manner to drive asthmatic IgE responses (112). In patients with severe asthma, NK cells expressed higher levels of CD69 and NKG2D in line with an activated phenotype. Despite higher activation status, NK cell ability to induce eosinophil apoptosis was impaired (113). IL-13 production by ILC2s was attenuated and NK cell-induced eosinophil apoptosis was greatly increased by lipoxin A4 (LXA4), a pro-resolving mediator negatively affected during severe allergic asthma (113, 114). Lacking efficiency in resolution of eosinophilic inflammation due to a lack of LXA4 production in severe asthma suggests another axis of interaction promoting pulmonary dysfunction of NK cell and ILC2 responses to inflammation (113). Collectively, multiple layers of regulation affect the localized activity and accumulation of ILCs in asthma, emphasizing the need to understand tissue signals that control ILCs to develop more targeted therapies.

### ILCs in COPD

Chronic obstructive pulmonary disease (COPD) is an inflammatory condition characterized by permanent and progressive loss of lung function, associated with smoking and exposure to noxious stimuli (115). ILC1s are increased in abundance in COPD patient lungs, correlating with smoking status and symptom severity (116). All helper ILC subsets localized with lymphoid aggregates in COPD lungs (116). IL-17 upregulation in end-stage COPD is implicated in lymphoid follicle neogenesis, and De Grove et al. found trends of elevated abundance of NCR^-^ ILC3s and IL-17A^+^ and IL-22^+^ ILCs in the lungs of COPD patients (66, 117). While this seems to support the involvement of ILC3s in COPD, data supporting a specific role for ILC3-derived IL-17 is lacking. Co-culture of expanded human lung ILC3s with mesenchymal stromal cells induced upregulation of ICAM-1 and VCAM-1, suggestive of LTi activity, contrasting with observations in *Rorc*^-/-^ and *Id2*^-/-^ mice that develop lung lymphoid follicles even in the absence of ILC3s/LTis (118, 119). Interestingly, a subset of Neuropilin1^+^ ILC3s were recruited to high endothelial venules in lung tissues of smokers and COPD patients in a VEGF-A-dependent manner, although the specific role for ILC3s in COPD development and pathogenesis remains unresolved (118).

Circulating NK cells from smokers and COPD patients express higher levels of CD57 and have greater cytotoxicity against autologous lung epithelium than non-smokers or smokers without COPD (120, 121). The increase in cytotoxicity was mirrored in a murine COPD model after cigarette smoke exposure, demonstrating that trans-presentation of IL-15Rα by lung DCs was required to prime high NK cell cytotoxicity against autologous epithelial cells (120). Interestingly, cigarette smoke exposure induces a sustained loss of ST2 expression on ILC2s, dramatically reducing their responsiveness to IL-33, despite increased IL-33 production in severe COPD (122). Conversely, smoke induces an upregulation of ST2 on NK cells, leading to IL-33-mediated activation of NK cells instead of ILC2s, explaining the increase in type 1 immunity despite elevation of the type 2-activating cytokine IL-33 (122). Paralleling this, elevation of circulating ILC1s with a strong inverse correlation to ILC2 abundance was observed in COPD patients, further suggesting a misguided immune activation and cytokine-driven ILC plasticity, similar to mechanisms observed in the response to murine influenza infections (89, 106). Stimulation of human blood-derived ILC2s with IL-12 promoted their trans-differentiation into ILC1-like cells accompanied by the downregulation of GATA3 and an upregulation of T-BET, increasing IFN-γ release while dampening IL-4 and IL-13 production (89, 106). These results collectively demonstrate that persistent lung inflammation and exposure to smoke leads to changes in the local ILC composition and function (**Figure 4D**).

## ILCs in the Skin

The skin is a barrier organ that employs immunological, microbial, and physiochemical mechanisms to protect the body from pathogens and harmful environmental factors. The skin is composed of three distinct layers: the epidermis (mainly comprised of keratinocytes), the underlying dermis, and the innermost subcutis. Tissue-resident and long-lived ILC subsets have been identified in mice and humans with varying proportions identified across studies (123–127). A more granular analysis of skin by layer revealed a predominant accumulation of ILC3s in the epidermis, ILC2s in the subcutis and comparable abundance of both subsets within the dermis in mice (128). This distribution has been attributed to the localized release of IL-7 and TSLP by either hair follicle keratinocytes or epithelial cells (128, 129). Mirroring murine models, human skin ILC2s can be activated by IL-25, IL-33, and TSLP, with high expression of IL-33 and TSLP during chronic skin inflammation (130–132).

**Figure 5:**
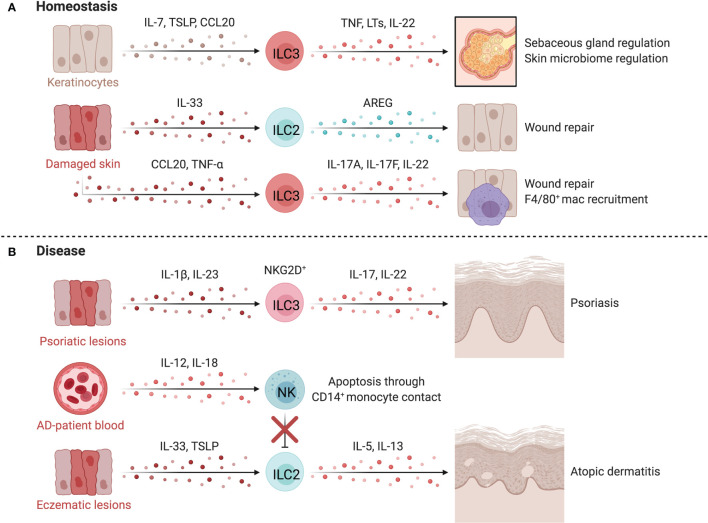
ILCs in the skin. At homeostasis **(A)**, ILCs are retained by IL-7 and TSLP released by hair follicle keratinocytes or epithelial skin cells. TNF-α^+^LTα1β2^+^CCR6^+^ ILC3s negatively regulate lipid-secreting sebaceous glands, regulating the skin microbiome, which can alter susceptibility to inflammatory conditions or affect tissue repair pathways. Upon tissue damage, injured epithelial cells release IL-33, inducing proliferation of skin-resident ILC2s. In mice, anti-CD90 depletion in *Rag1*^-/-^ mice delays wound healing, suggesting a role for ILC2s in promoting epithelial repair *via* AREG. The epithelium also produces TNF-α downstream of damage-induced Notch signaling in keratinocytes, recruiting CD4^+^NKp46^low/-^ ILC3s that participate in wound closure. TNF-α driven release of CCL20 and CXCL13 by keratinocytes recruit ILC3s which facilitates the recruitment of F4/80^+^ reparative macrophages. In disease **(B)**, psoriatic lesions in mice accumulate IL-17^+^ IL-22^+^ NCR^+^ ILC3s which is mirrored by the increase of CD56^+^RORγt^+^ ILC3s in both inflamed and non-inflamed skin of psoriasis patients. ILC2s promote atopic dermatitis (AD) when activated by epithelial-derived TSLP and IL-33 in inflammatory lesions. Dermal NK cells are decreased in AD and prone to apoptosis through contact with CD14^+^ monocytes. NK cells are proposed to regulate ILC2 abundance in AD, as therapeutic expansion of NK cells lowers ILC2 counts and improves disease scores in an AD mouse model. Created with Biorender.org.

ILCs regulate essential homeostatic functions of the skin (**Figure 5A**). For example, murine ${\text{TNF}}^{+}{\text{LT}\alpha }_{1}\beta_{2}^{+}{\text{CCR}6}^{+}$ ILC3s negatively regulate the size of the lipid-secreting sebaceous glands, while the differentiation, proliferation, and expression of antimicrobial proteins by keratinocytes depends on IL-22 stimulation from ILC3s or epidermal T cells (128, 133). These interactions regulate the skin microbiome which can alter susceptibility to inflammatory disorders, impact repair pathways and influence host defense (128, 133, 134).

### ILCs in Wound Healing

ILCs directly influence skin repair after damage. Murine skin-resident ILC2s activated by IL-33 from injured epithelial cells proliferate at sites of injury while anti-CD90 depletion of ILC2s in *Rag*1^-/-^ mice delays wound healing (135). CD4^+^NKp46^low/-^ ILC3s are recruited by damage-induced CXCL13 and CCL20 and promote wound closure *via* IL-17A, IL-17F and IL-22 and indirectly through CCL3-mediated macrophage recruitment (136). Comparable findings were observed in *IL-22*^-/-^ mice, where deficiency in IL-22 impaired keratinocyte proliferation, impeding repair (137). These results support a role for ILC2s and ILC3s in regenerative remodeling of the skin, yet research is needed to translate animal findings to humans and to define the differential impacts of ILCs and T helper cells (138).

### ILCs in Psoriasis

Psoriasis is a chronic inflammatory skin disease that manifests as red scaly plaques caused by hyperproliferation of keratinocytes downstream of excessive repair pathways (139). Elevated IL-17 levels and Th17-associated gene expression signatures are found in psoriatic lesions and mouse models, implicating IL-17 and IL-22 in pathogenesis (140–142). IL-22- and IL-17-producing NCR^+^ ILC3s and CD56^+^RORγt^+^ ILC3s are enriched in inflamed and non-inflamed skin of psoriasis patients (**Figure 5B**) (123, 124, 126). ILC3s in inflamed lesions express higher NKG2D, which likely interacts with elevated MICA on keratinocytes (143). Anti-TNF treatment reduced circulating ILC3s in patients, corresponding with a decrease in inflammatory lesions (124). Further, ILC3-derived IL-22 induces an upregulation of MHC-II on keratinocytes, which promotes T cell polarization and skin inflammation, demonstrating a key circuit mediating skin inflammation (144). Skin ILC2s are also capable of driving T cell activation directly by presenting lipid antigens in a CD1a-dependent manner, leading to local activation of T cells in response to dermal bacteria (145).

There is limited and sometimes conflicting evidence for the role of NK cells in psoriasis. Studies have indicated that circulating NK cells are reduced in psoriasis patients (146, 147), or that no change was observed compared to healthy controls (148, 149). Within psoriatic plaques, Ottaviani et al. observed CD56^+^CD16^-^ NK cells that co-expressed CD161, NKG2A, and CD69 (150). Supernatants from culturing these NK cells activated keratinocytes, increasing MHC-I, ICAM-1 and HLA-DR expression, along with CXCL10 and CCL5 secretion (150). These chemokines induced migration of skin-derived NK cells, supporting NK cell-keratinocyte cross-talk in psoriatic inflammation (150). NK cells appear to be hypofunctional in psoriasis, with reduced degranulation and IFN-γ potential (146, 149). The role of helper type 1 ILCs is even less defined, however expansion of ILC1s was observed in psoriatic lesions (126).

### ILCs in Atopic Dermatitis

Atopic dermatitis (AD) is a common inflammatory skin disorder characterized by high levels of IL-4, IL-5 and IL-13 (151, 152). AD skin lesions are enriched for skin-resident ILC2s, which are activated by TSLP or IL-33, promoting type 2 inflammation (132, 153, 154). This is supported by murine models where anti-CD90 and anti-CD25 depletion of skin ILC2s in T and B cell deficient *Rag1*^-/-^ mice attenuated dermatitis symptoms (153). Interestingly, KLRG1 ligation by E-cadherin reduces IL-5 and IL-13 production by human ILC2s, implicating dysregulation of parenchymal-ILC interactions in AD where E-cadherin levels are canonically downregulated on keratinocytes and ILC2s have elevated KLRG1 expression (132).

ILC3s have also been implicated in the pathogenesis of AD. Circulating ILC2s and ILC3s are elevated in AD patients, and increased IL-17 levels are apparent during acute disease (154, 155). Using several AD models, Kim et al. demonstrated AD lesions had increased numbers of IL17A^+^ ILC3s, which induced IL-33 release by keratinocytes and fibroblasts, promoting type 2 responses and exacerbating disease in mice (154). Further supporting a role for ILC3s in AD, ILC2s and ILC3s were elevated in AD lesions, with AHR^+^ ILC3s representing the most abundant subset. ILC3s in AD lesions were frequently surrounded by T cells, suggesting cellular interactions between ILC3s and T cells in AD (126).

NK cells are also altered in AD and are prone to apoptosis *via* a CD14^+^ monocyte-driven, contact-dependent mechanism, aligning with observed reductions in peripheral NK cell abundance in AD (146, 152, 156). Mack et al. found particularly reduced levels of circulating mature CD56^dim^CD16^+^ NK cells with high expression of KIRs and CD57 in patients with moderate-to-severe AD (152). A regulatory circuit between NK cells and ILC2s is supported by three lines of evidence: NK cell recovery occurring after IL-4 blockade; ILC2 accumulation in AD lesions of NK cell-deficient mice; and NK cell recovery and activation after IL-15 superagonist treatment leading to reduced ILC2 levels and disease scores in an AD model (152). Thus, cross-talk between ILC subsets may underlie the development and severity of AD (**Figure 5B**).

## ILCs in the Intestine

**Figure 6:**
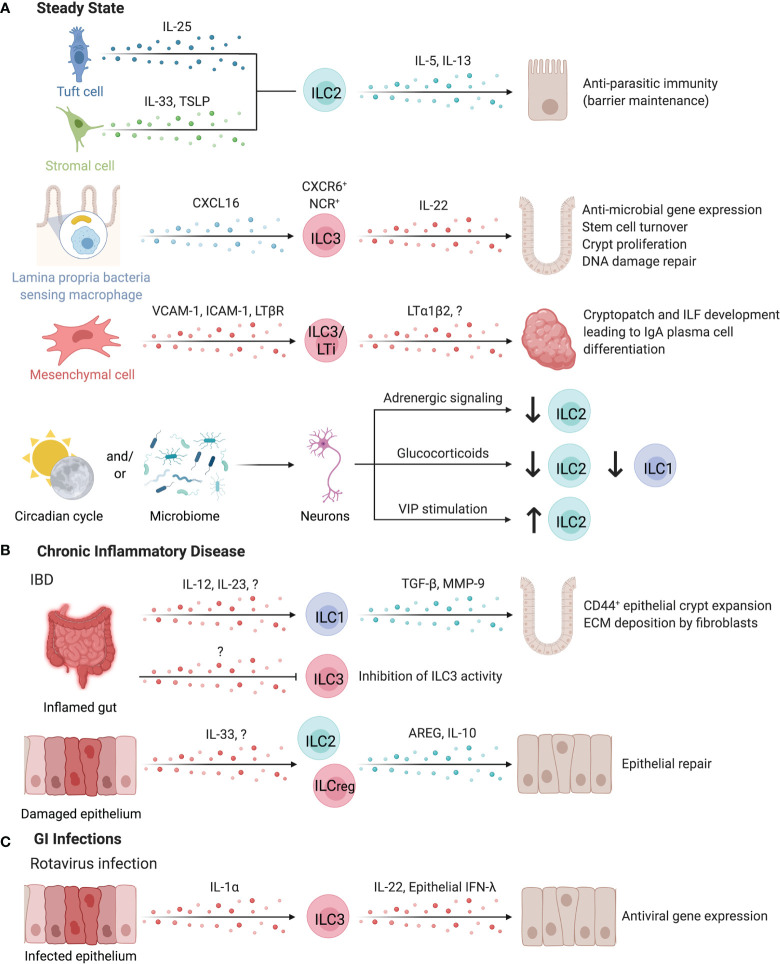
ILCs in the intestines. ILCs have important roles in maintaining intestinal homeostasis **(A)**. ILC2s can be activated by both IL-25-producing tuft cells or IL-33 and TSLP-secreting stromal cells to promote anti-parasitic immunity in the intestines. ILC2s secrete IL-5 and IL-13 to promote host defense through parasite expulsion. Similarly, microbiota-sensing CX3CR1^+^ macrophages position IL-22-secreting CXCR6^+^ NCR^+^ ILC3s in the lamina propria *via* CXCL16. IL-22 supports anti-microbial gene expression in Paneth cells and promotes stem cell turnover, crypt proliferation, and DNA damage repair. Interaction of ILC3/LTi surface LTα_1_β_2_ and LTBR on mesenchymal cells leads to the upregulation of VCAM-1 and ICAM-1, resulting in the formation of cryptopatches and ILF. These tertiary lymphoid structures support the differentiation of IgA-producing plasma cells to promote barrier defense and host-microbiota mutualism. Clock genes and circadian cycles, modulated through feeding and the microbiota drive important homeostatic neuro-immune interactions in the gut. Disruption of circadian regulation alters ILC3 function, abundance, and trafficking into the intestines while negatively regulating ILC2s through adrenergic signaling. Glucocorticoids or vasoactive intestinal peptide further control ILC1 and ILC2 responses. **(B)** In chronic inflammatory disease such as intestinal bowel disease (IBD), the inflamed gut induced TGF-β and Matrix metalloproteinase 9 production by ILC1s leading to the expansion of epithelial crypt cells and extracellular matrix deposition by fibroblasts, exacerbating fibrosis. In contrast, pro-tolerogenic ILC3 functions including the release of GM-CSF, IL-2 or the expression of MHC-II are impaired in IBD patients, suggesting an anti-inflammatory role for ILC3s. IL-10 producing regulatory ILC2s or ILCregs may also suppress intestinal inflammation. **(C)** Enteric infection by Rotavirus induces epithelial-derived IL-1α to promote ILC3 production of IL-22, which synergizes with epithelial IFN-λ to promote the induction of antiviral responses in intestinal epithelial cells. Created with Biorender.org.

The intestine is the largest mucosal surface in the human body and faces unique challenges. As a barrier surface, immune function in the intestine must balance tolerance and control of commensal microbes with protection from pathogens. Among immune residents of the intestine, ILCs have key roles in sustaining gut barrier integrity, repair, immune homeostasis, and host defense (**Figure 6A**). ILC distribution along the human intestine was reported by Simoni et al. and Yudanin et al. (67, 127). In line with observations made in mice, both groups demonstrated the presence of NK cells, ILC1s, ILC2s, and ILC3s across the intestinal tract, with predominance of ILC1s and ILC3s (67, 127, 157). NK cells are low in abundance and mainly CD56^bright^ with distinct surface marker expression (64) (**Figure 2**). Intestinal ILC1s are heterogeneous, including a population of CD103^+^ ILC1s located in the epithelium, and CD127^+^ ILC1s residing in the lamina propria (LP) (158, 159). ILC3 subsets also localize within distinct microanatomic compartments of the gut epithelium/isolated lymphoid follicles(ILFs)/LP, but it remains to be shown if a similar distribution applies to humans (160, 161).

Intestinal ILCs promote host immunity against pathogenic and commensal microbes through interactions with sentinel immune and tissue cells. For example, murine ILC2s activated by IL-25-producing tuft cells or IL-33- or TSLP-secreting stromal cells promote anti-parasitic immunity, while DC-derived IL-33 promotes regulatory T cell (Treg) responses, suppressing anti-parasitic immunity (4, 24, 162–164). Myeloid cells, especially CXCL16-producing CX3CR1^+^ macrophages are critical for sustaining lamina propria-resident CXCR6^+^ NCR^+^ ILC3s as a major source of IL-22 in the intestinal LP (160). These ILC3s support IL-22-dependent intestinal epithelial anti-microbial gene expression, stem cell turnover, crypt proliferation, and DNA damage repair (160, 165–168).

While ILC3-derived IL-22 protects the intestinal epithelium against genotoxic stress, risk-associated single nucleotide polymorphisms have been identified within *Il22* and the IL-23 signaling pathways as a driver of colorectal cancer in patients (165, 169, 170). Nevertheless, ILC3-derived IL-22 and LTα positively alter the glycosylation activity of epithelial cells, supporting glycan-scavenging intestinal commensal microbes and balanced host-microbe interactions and providing protection from infection (171, 172). sLT, expressed by human and mouse LTis, is essential to initiate the development of cryptopatches (CPs) and ILFs in the gut (11, 173). These tertiary lymphoid tissues support the differentiation of IgA-producing plasma cells to promote barrier defense (174, 175). Mouse CP and ILFs contain a unique subset of DCs that require LTβR signaling for their development. These DCs released IL-22 binding protein, which in turn alter intestinal epithelial IL-22R signaling and lipid transport (176).

Tregs have key functions in inducing tolerance to luminal antigens (177). IL-2 and GM-CSF-producing ILC3s directly and indirectly support the generation of Tregs in the healthy murine gastrointestinal tract, upon stimulation by microbiota-sensing IL-1β-producing macrophages. The cooperation and reciprocal crosstalk between macrophages, DCs, and ILC3s supports Treg homeostasis and T cell immunity against orally ingested antigens (177–179). MHC-II expression on murine ILC3s has been demonstrated to regulate T cell responses to microbial antigens *via* a mechanism analogous to negative selection in the thymus (180, 181). Lehmann et al. reported organ-specific expression levels of MHC-II on murine ILC3s and demonstrate that microbiota-induced IL-23 stimulation of ILC3s reversibly downregulated their MHC-II expression (182). Noteworthy, Rao et al. reported an accumulation of HLA-DR^+^ ILC3s in T cell-rich areas of colorectal cancers suggesting antigen-presenting capacity of ILC3s in humans as well (183). Together, this suggests that ILC3s both positively and negatively regulate T cell immunity dependent on microenvironmental signals.

Several environmental factors regulate murine intestinal ILC abundance. The metabolite-sensing Ahr is highly expressed by ILCs in the gut, with an important role in sustaining ILC3s and promoting IL-22 production (165, 184, 185). In contrast to ILC3s, gut ILC2 function is suppressed by Ahr signaling, suggesting a role for Ahr ligands in regulating the balance of intestinal ILC subset abundance (186). A similar divergent stimulation between ILC2s and ILC3s has been reported for other dietary components (187, 188). Microbial short chain fatty acids (SCFAs) differentially affect mouse ILCs in a subset- and location-specific manner, generally promoting ILC3 proliferation and IL-22 production while inhibiting ILC2 expansion (189–191). Free Fatty Acid Receptor 2 (Ffar2) acts as a SCFA receptor, and agonism leads to ILC2 proliferation, yet SCFA feeding leads to contraction of ILC2 abundance, suggesting the involvement of several receptors in coordinating the response to microbial fermentation products (191). This along with reports of age and body-mass index-associated alterations in the abundance of ILC subsets suggests age and metabolism-dependent regulation of intestinal ILCs in humans (67).

Cholinergic neurons in the gut and lung of mice produce neuromedin U in response to helminth challenge, which stimulates ILC2 proliferation and production of IL-4 and IL-13 in an IL-33-independent manner (192). The neuromedin U receptor does not appear to be expressed by other hematopoietic cells besides ILC2s at significant levels (192). In humans, the *NMUR1* transcript was detected in intestinal ILC2s, yet direct evidence for this ILC2-neuronal interaction in humans is lacking (192). Other modalities where the nervous system regulates ILCs includes negative regulation of ILC2s by adrenergic signaling, glucocorticoid dampening of ILC1 and ILC2 responses, VIP stimulation of ILC2s, and ILC3 co-localization with neurons in enteric CPs, as detailed in a review by Klose and Artis (3). Interestingly, circadian light-dark cycles regulated neuron-immune interactions and intestinal ILC3-specific gene expression through diurnal oscillations of *Rorc*, *Il17a*, and *Il22*, while disruption of ILC3 circadian regulation altered their function, abundance, and trafficking in the murine intestine (2, 193–195). Interestingly, the gut microbiota contributed to control of this circuit, as antibiotic treatment partially restored ILC3 abundance and constrained cytokine production in circadian-disrupted mice (195). In mice, VIP promotes ILC3 intestinal recruitment and maintains expression of gut-homing receptor CCR9 (196). Talbot et al. reported a feeding-induced inhibition of ILC3s by VIPergic neurons, regulating mucosal immunity by dampening IL-22-induced antimicrobial peptide production in exchange for enhanced absorptive capacity of the intestinal epithelium marked by increased fatty acid transporter (*Fabp2*) expression (197). This contrasts with findings by Seillet et al. that VIP stimulation increased IL-22 production by enteric ILC3s, although the reason for these conflicting results is unclear, suggesting complex signals regulate intestinal ILC3 activity (198). Together, intestinal ILC3s are regulated by a complex circadian network involving light-dark cycles, microbial signals, and nutrient-driven neuronal regulation. Of note, the production of IL-5 by murine ILC2s was also circadian regulated (198).

### Chronic Inflammatory Diseases

Chronic inflammation of the intestinal tract is a hallmark of inflammatory bowel disease (IBD) and fosters a local cytokine milieu that promotes differentiation of ILC1s (199). ILC1 expansion in inflamed intestinal tissue is location-specific, with greater expansion of LP-resident CD127^+^ ILC1s versus intraepithelial ILC1s in Crohn’s disease (CD) patients (158, 159, 200, 201). Specific expansion of CD127^+^CD94^+^Granulysin^+^ ILC1s is observed in the inflamed LP of CD patients (202). With elevated secretion of TGF-β and MMP9, mouse ILC1s facilitate the expansion of CD44^+^ epithelial crypt cells and extracellular matrix deposition by fibroblasts, collectively supporting matrix remodeling and epithelial proliferation that may exacerbate inflammation-associated fibrosis (**Figure 6B**) (203). In contrast, ILC3 abundance and homeostatic functions in circadian oscillation, production of IL-2, and expression of MHC-II were critically impaired in IBD patients, supporting anti-inflammatory contribution of ILC3s (178, 180, 193, 195). ILC3 secretion of IL-22 is enhanced by G Protein-Coupled Receptor 34 (GPR34) recognition of lysophosphatidylserine from apoptotic neutrophils, further supporting a role of ILC3s in sensing intestinal injury and initiating repair responses (204). However, ILC3s may contribute to intestinal inflammation under permissive circumstances (205). Further, destabilizing RORγt expression promoted the differentiation of ILC3s into ILC1/ex-ILC3 in mice and humans and correlated with intestinal IBD-like inflammation (206, 207). Interestingly, this differentiation was not static, but was regulated by the myeloid cytokine milieu in the intestinal tract (159). Counterbalancing the elevated type 1 and type 3 immunity reported in IBD, ILC2-derived AREG was sufficient to reduce DSS-induced damage in mice by promoting epithelial integrity and mucus production (162). Bando et al. further identified murine ILC2s as a dominant source of IL-10 in the intestine, while Wang et al. identified a distinct subset of IL-10-producing regulatory ILCs in humans and mice, supporting that IL-10 producing ILC2s or ILCregs may suppress intestinal inflammation (**Figure 6B**) (13, 17). Targeting ILC3-to-ILC1 plasticity, ILC1 activation, and ILC3 abundance may be a promising approach to restore intestinal immune homeostasis under chronic inflammatory conditions (208–211).

### Gastrointestinal Infection

ILCs play a critical role in the response to intestinal pathogens in humans, highlighted by cases of deficiency in *RORC* resulting in severe mucosal fungal and bacterial infections (212). Along this line, susceptibility to infections by enteric extracellular pathogens are increased in the absence of IL-22 or GM-CSF, highlighting a critical role for ILC3-associated cytokines in barrier defense (213–216). Mouse ILC3s and ILC1s/ex-ILC3s promote antimicrobial responses *via* surface lymphotoxin-mediated differentiation of goblet cells and IFN-γ-induced production of mucins, further emphasizing the synergistic actions of ILC1s and ILC3s that require underlying microbial recognition and activation by myeloid cells (207, 217–219). Whether this permits discrimination of commensal and pathogenic microbes requires further investigation (219). In response to mouse enteric rotavirus infections, epithelial IL-1α induced ILC3-derived IL-22 which synergized with epithelial IFN-λ, promoting the induction of antiviral gene expression in intestinal epithelial cells, limiting viral replication and tissue damage (**Figure 6C**) (220). While ILC3s can promote antiviral immunity, they experience cytokine-dependent depletion in the intestinal tract of HIV^+^ human and SIV^+^ non-human primates, altering epithelial permeability and homeostasis (221–223). Collectively, ILCs promote intestinal barrier defense against enteric bacterial, fungal, and viral infections by exerting cytokine or cell contact-dependent effects on intestinal epithelial cells.

Enteric parasites and worms constitute a major global health burden. Murine NK cell recruitment to the intestine early after helminth infection does not affect parasite burden but limits the tissue damage induced by infection (224). Experimental models of worm infections revealed the importance of ILC2s and ILC2-derived cytokines in intestinal host defense in mice (225–227). For example, IL-13 from murine ILC2s promoted tuft and goblet cell differentiation from crypt progenitors, contributing to epithelial remodeling and worm expulsion in the characteristic “weep and sweep” response (4, 228). ILC2s actively promoted Th2 cell responses *via* MHC-II and co-stimulatory molecules, partially acquired through trogocytosis, while T cell-derived IL-2 activated ILC2s for efficient helminth expulsion in mice (229). Further, ILC2s are activated by acetylcholine and upregulate ChAT to produce acetylcholine in response to helminth infection, supporting efficient helminth expulsion through a potential autocrine signaling mechanism (230). The activation of ILC2s following worm infection could be blunted through parasite-derived, bio-active components interfering with the IL-33-ST2 axis (231). Intriguingly, helminth infection changed the global distribution and activation of murine ILC2s through the induction of S1PR1-dependent egress of gut ILC2s and accumulation in the lungs, suggesting a coordinated response to protect distal body sites targeted by helminth infection (72, 232).

While the fetal and adult human intestine hosts a population of ILC2s capable of releasing type 2 cytokines following stimulation with IL-2, IL-25, and IL-33, their role during human parasitic infections has not been well detailed (104). Lack of sample availability has hampered investigation of intestinal ILC abundance and function of worm infected patients (233). To date only two studies analyzed ILCs in worm infected patients. Nausch et al. observed a reduced frequency of ILC2s in children infected with Schistosoma, while Boyd et al. observed an increase in circulating c-kit^+^ ILCs and elevated IL-13 secretion in adult patients with filarial infections, suggesting heterogeneity in ILC responses dependent on age and/or helminth species (234, 235).

Collectively, intestinal ILCs support host immunity, barrier defense and tissue repair during infection and homeostasis, but also may perpetuate inflammation under permissive microenvironmental conditions.

## ILCs in the Liver

The liver is critical for metabolism and blood detoxification. Constant exposure to an array of antigens and microbial products within liver sinusoids promotes tolerance to predominantly harmless antigens (236, 237). The liver contains a large proportion of innate immune cells such as Kupffer cells (specialized macrophages), inflammatory and non-inflammatory macrophages, NKT cells, NK cells and ILCs (238–240). These innate lymphocytes influence the activation and function of the various adaptive immune populations that include αβ T cells, γδ T cells and B cells, as well as parenchymal cells within the liver niche.

In humans, CD56^bright^CD16^-^ NK cells comprise 50% of all liver NK cells (241). These NK cells express CD69, CCR5 and CXCR6, but not SELL or CCR7, and are localized to sinusoids by CCL3, CCL5, and CXCL16 produced by Kupffer cells, T and NK cells, and endothelial cells, respectively (241, 242). NK cells in healthy liver of deceased donors highly express *EOMES*, *CD7*, *KLRD1*(CD94), *GZMK*, *NCR1*(NKp46) and *NCAM1*(CD56), and lowly express *FCGR3A*(CD16) and *ITGA1*(CD49a) (240). Although CD49a^+^ NK cells akin to murine liver-resident NK cells have been identified in humans, they represent only a small subset of human liver-resident NK cells, while lack of CD49e protein expression differentiated human liver-resident NK cells from conventional (cNK) cells (243, 244). CD49a^+^CD16^−^ NK cells in liver have a transcriptional program consistent with cytotoxic activity and exhibited antigen-specific killing of autologous targets presenting viral or metal antigens (245). Notably, a donor-derived EOMES^hi^ tissue-resident NK cell population persisted in the liver up to 13 years post-transplant in a study of HLA-mismatched liver transplants (246). This NK cell population had a phenotype consistent with those reported in transcriptomic studies of healthy human liver (240–243).

While group 1 ILCs are the most abundant hILC population in human liver, NCR^+^ and NCR^-^ ILC3s and ILC2s are also present (247). Liver ILC2s are CRTH2^+^CD161^+^CD69^+^ and highly express fibronectin-binding VLA-5, laminin-binding VLA-6, and the chemokine receptor CCR6 (247). In contrast to mice, only 10% of intrahepatic human ILC2s express the IL-33 receptor ST2, and primarily produce IL-13 and AREG, with very little IL-5 (247).

### ILCs in Viral Hepatitis

**Figure 7:**
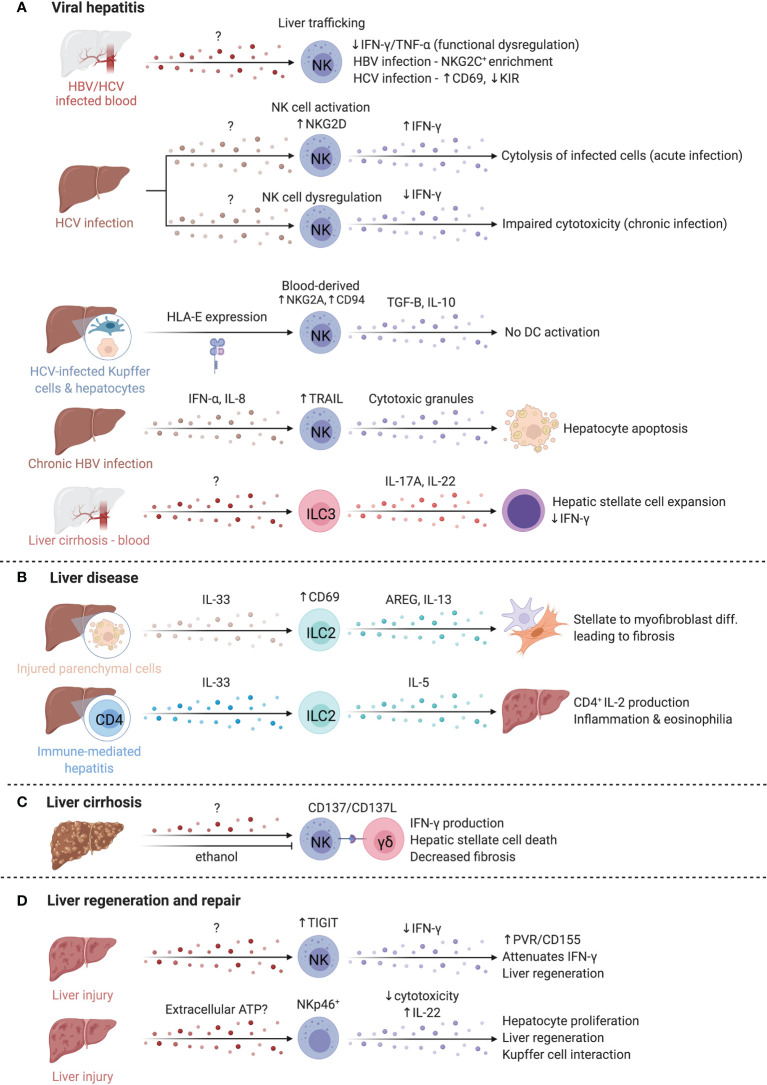
ILCs in the liver. The liver is occupied by a variety of ILCs that play diverse roles in viral hepatitis, liver disease, liver cirrhosis, and liver regeneration and repair. **(A)** Hepatitis C (HCV) and Hepatitis B (HBV) are key inducers of liver inflammation and cirrhosis and lead to the development of hepatocellular carcinoma. The abundance of NK cells in the blood of both HBV/HCV infected patients is reduced, suggesting elevated homing to the liver where functional deficiencies like impaired IFN-γ and TNF-α production are reported. NK cells in chronic HBV and HCV infected patients adopt distinct phenotypes that manifest in an enrichment of NKG2C-expressing NK cells or altered CD69 and inhibitory KIR expression. In contrast, acute HCV infection promotes elevated NKG2D and IFN-γ expression while IFN-γ is reduced in chronic infections. HCV-infected hepatocytes and Kupffer cells express higher levels of HLA-E that boost TGF-B and IL-10 production by NK cells through NKG2A and CD94. CD56^bright^ NK cells in chronic HBV infection facilitate TRAIL-dependent hepatocyte death. ILC3s separately support hepatic stellate expansion and counteract IFN-γ. **(B)** In liver disease, ILC2s are increased and activated, driving liver fibrosis *via* AREG and IL-13. Similarly, in immune-mediated hepatitis, ILC2s were expanded and produced high levels of IL-5, recruiting eosinophils, and driving inflammation. **(C)** In liver cirrhosis, crosstalk between NK cells and γδ T cells through the CD137-CD137L axis enhanced cytotoxicity of NK cells against HSCs. Alcohol exacerbates fibrosis chronically, but also attenuates NK-mediated cell killing, and reduced NKG2D, TRAIL and IFN-γ expression on NK cells. **(D)** NK cells upregulate TIGIT while hepatocytes upregulated the ligand PVR/CD155, attenuating IFN-γ production and promoting liver regeneration. Finally, extracellular ATP is elevated after liver injury and regulates regeneration in the liver *via* NKp46^+^ NK cells. Created with Biorender.org.

NK cells are implicated in both Hepatitis C (HCV) and Hepatitis B (HBV) infections, which are major causes of liver inflammation and cirrhosis, leading to development of hepatocellular carcinoma (248) (**Figure 7A**). Peripheral NK cell abundance is reduced in both HCV- and HBV-infected patients, with reduced IFN-γ and TNF-α potential particularly in HBV, suggesting functional dysregulation (249). Cytotoxic impairment is associated with chronic infection establishment, while acute HCV infection induces NK cell activation, including increased NKG2D expression and greater capacity for cytotoxicity and IFN-γ production (250). Despite shared dysregulation, NK cell phenotype differs between chronic HBV and HCV; an enrichment of NKG2C^+^ NK cells are observed in HBV, whereas increased CD69 expression and decreased inhibitory KIR expression are observed in HCV (249). Differences in NK cell KIR and HLA allele expression may differentiate infections that are self-limited versus those that become chronic; KIR2DL3 and HLA-C1 expression is reported to be protective in HCV infection (251, 252). Weaker inhibitory signals by HLA-C1 may allow for increased NK cell activation and viral clearance (251, 252). In agreement, degranulation marker CD107a was increased on NK cells with KIR2DL2/3 and was highest in those with self-limiting infections (250). Engagement of HLA-E with elevated NKG2A and CD94 receptors on NK cells of HCV-infected individuals results in TGF-β and IL-10 production and impaired ability to activate DCs for virus-specific T cell responses, in line with findings that hepatocyte and Kupffer cell HLA-E expression correlates with HCV severity (253, 254). Of note, intrahepatic CD56^bright^CD16^-^ NK cell abundance correlates with better liver function and lower disease scores in HCV-positive patients undergoing liver transplantation (255).

In chronic HBV, circulating and intrahepatic NK cells highly express TRAIL and CD69, especially the CD56^bright^ subset (256). Elevated IFN-α and IL-8 upregulate TRAIL expression on NK cells and TRAILR-2 expression on hepatocytes, respectively, suggesting TRAIL-dependent targeting of hepatocytes by CD56^bright^ NK cells mediates damage during chronic HBV flares (256). Notably, HBV-specific T cells also have high expression of TRAIL-R2 and are susceptible to targeting by NK cells, supporting a role for NK cells in regulating anti-HBV T cell responses (257).

Comparatively little is known about the role of human hILCs in hepatitis infections. Increased hILCs were reported in the circulation of patients with chronic HBV (258, 259). HBV-related cirrhosis progression correlated with IL-17A and IL-22 production by ILC3s, suggesting ILC3 promotion of fibrosis, likely in part due to IL-22-mediated suppression of anti-fibrotic IFN-γ (**Figure 7A**) (259). While HCV/HBV do not infect mice, other viral hepatitis models provide some context into ILC viral responses in the liver more generally. Hepatic ILC3s produce IL-17A/F alongside γδT cells to promote antiviral T cell responses and inflammation early after infection (260). At later timepoints post-infection, ILC2s induce immunosuppressive neutrophils *via* IL-13 to limit T cell damage (261). This suggests that hepatic ILCs may have time-dependent roles to balance viral clearance and tissue protection.

### ILCs in Liver Fibrosis

Liver disease is characterized by fibrogenesis of the liver, driven by type 2 immunity, with an implication for ILC2 activity (**Figure 7B**) (262). Hepatic stellate cells become activated and transdifferentiate into myofibroblasts that produce copious extracellular matrix proteins, driving fibrosis and loss of function resulting in cirrhosis (263). Patients with cirrhosis have elevated serum IL-33 and increased intrahepatic ILC2s, correlating with disease severity (247, 264, 265). Expansion of ILC2s and activation by IL-33 from damaged parenchymal cells results in IL-13 production driving fibrotic gene expression in hepatic stellate cells in fibrosis models, or IL-5 production with resultant hepatic inflammation and eosinophilia in immune-mediated hepatitis models (265, 266). While both effects are IL-13-dependent, additional signals which influence IL-5 versus IL-13 dominant responses by ILC2s are unknown. Interestingly, liver ILC2s present antigen to CD4^+^ T cells which produce IL-2 to sustain ILC2 expansion (267). High levels of IL-6, linked to liver regeneration, were produced by IL-33-activated liver ILC2s, indicating that ILC2s may have a dual roles in immune-mediated liver disease (267).

Intrahepatic human CD49a^+^ NK cells are expanded in cirrhotic livers (268). CD49a^+^CD25^+^ NK cells positively correlate with serum alanine aminotransferase, linking CD49a^+^CD25^+^ NK cells to liver inflammation (268). Conversely, liver NK cells dampen fibrosis by killing activated hepatic stellate cells in an NKG2D- and TRAIL-dependent manner, while IFN-γ reduces hepatic stellate cell activation and matrix protein deposition (269, 270). CD137-CD137L crosstalk between NK cells and γδ T cells enhances NK cell cytotoxicity (**Figure 7C**) (271). Chronic alcohol consumption exacerbates fibrosis, and ethanol attenuates NK cell cytotoxicity towards hepatic stellate cells by reducing NKG2D, TRAIL, and IFN-γ expression, suggesting immunological and environmental mechanisms of NK cell regulation (272).

### ILCs in Non-Alcoholic Liver Disease

Non-alcoholic fatty liver disease (NAFLD) is the most common non-infectious chronic liver disease and can develop into non-alcoholic steatohepatitis (NASH) and progress to cirrhosis (273). NK cells are elevated in liver biopsies of NAFLD and NASH patients, with greater than two times increased NK cell abundance in NASH compared to NAFLD (274). *NKG2D* and *TRAIL-DR5* transcript levels also have higher expression in NASH (274). Upregulation of *MIC-A/B* positively correlates with disease score and degree of fibrosis, suggesting NK cell engagement with MIC-A/B stress ligands could be a key factor in NASH development (274). In agreement, circulating NK cells from NASH patients had higher NKG2D expression (275). Depletion of IFN-γ-producing NKp46^+^DX5^+^ NK cells in a NASH mouse model altered macrophage phenotype, suggesting that IFN-γ from NK cells reduces fibrosis by polarizing macrophages away from a TGF-β^+^ pro-fibrotic phenotype (276). Additional studies are required to delineate the mechanisms that control whether NK cells limit or promote fibrosis.

While fewer studies have focused on hILCs, ILC3s appear to mitigate NAFLD. High fat diet increases ILC3 abundance in mice, while deficiency of ILC3s leads to liver fibrosis and an increase of pro-inflammatory gene expression with concomitant accumulation of saturated fatty acids (277).

### ILCs in Liver Regeneration and Repair

The liver is uniquely capable of self-regeneration, including regenerating entire lobes after resection. Group 1 ILCs interact with injured tissue and influence regenerative capacity (**Figure 7D**). In models where NK cells are pre-activated by viral infection or TLR3 agonism to produce higher levels of IFN-γ, as well as in aged livers that have elevated IFN-γ signaling, regeneration is impaired (278, 279). NK cells upregulate T cell immunoreceptor with Ig and ITIM domains (TIGIT) while hepatocytes upregulate the ligand PVR/CD155, attenuating IFN-γ production and promoting liver regeneration (280). Mouse NKp46^+^ cells co-localized with F4/80^+^ cells in liver sinusoids, and NKG2D blockade abrogated regeneration, suggesting NKG2D-mediated crosstalk with Kupffer cells regulates regeneration (281). Extracellular ATP is elevated after injury and regulates liver regeneration (281, 282). ATP limits NK cell cytotoxicity while antagonism of ATP-receptor P2X1 reduces IL-22 production by group 1 ILCs in a murine liver resection model, resulting in dampened hepatocyte proliferation and elevated hepatocellular injury and stress (281, 283). Taken together, extracellular ATP released after resection may dampen NK cell cytotoxicity and promote IL-22 production to modulate time-dependent group 1 ILC functions supporting liver regeneration. Future studies that characterize marker expression in greater detail may clarify whether the cells identified were also inclusive of CD56^+^ ILC3s or were NK cells or ILC1s that converted to ILC3s.

## ILCs in the Kidney

**Figure 8:**
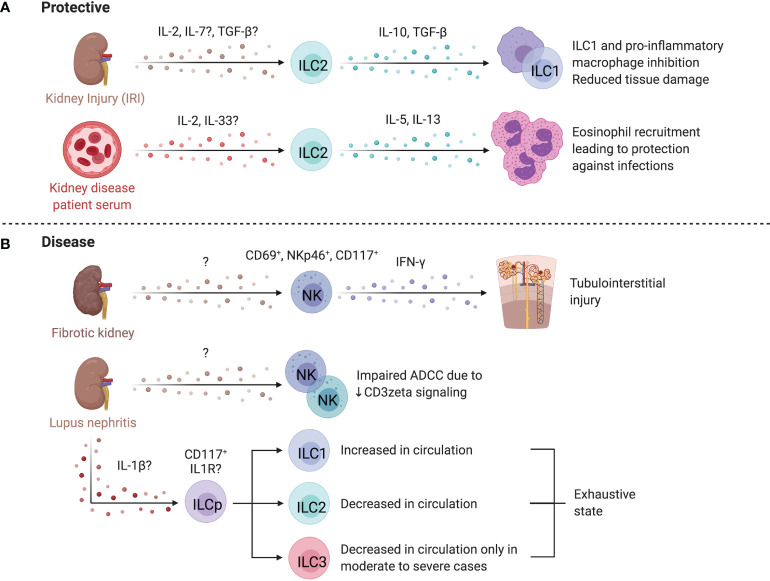
ILCs in the kidney. **(A)** Mouse and human studies support ILC2s may limit kidney injury. Administration of IL-25 and IL-33 in a humanized mice model attenuates IRI, and in conventional mouse models promotes a Th2 response and M2 macrophage polarization resulting in decreased tissue damage post ischemic injury. ILC2s may also be protective in end-stage renal disease (ESRD), where circulating ILC2 abundance, proliferation, and cytokine release increases. IL-2 is proposed to facilitate this ILC2 expansion *via* STAT5 and protect against infections through eosinophil support. **(B)** CD56^bright^ NK cells are more abundant in fibrotic kidney tissue where they upregulate CD69 and co-express NKp46 and CD117, producing the majority of IFN-γ, implying a role in driving inflammation and fibrosis. In lupus nephritis (LN), scRNAseq revealed two distinct NK cell subsets – a CD56^dim^CD16^+^ blood-derived and tissue-resident CD56^bright^CD16^-^ population. Both NK cells showed impaired antibody-dependent cell cytotoxicity because of dampened signaling efficiency by NKp30 and NKp46. LN patients further displayed elevated ILC1s and decreased ILC2s, while patients with moderate to severe disease showed an additional decrease in ILC3s. Created with Biorender.org.

Kidneys perform essential functions of filtering blood, excreting waste, and regulating the body’s fluid and electrolyte balance. ILCs have been found to contribute to acute and chronic kidney diseases, with protective (**Figure 8A**) and pathological (**Figure 8B**) functions in IRI, kidney disease, and lupus nephritis, however, their role in the steady state remains poorly described.

### ILCs in Chronic Kidney Disease

End-stage renal disease (ESRD) is associated with high morbidity and mortality, often associated with infections (284). Circulating ILC2 abundance, proliferation, and IL-5/IL-13 production is higher in patients with ESRD versus healthy controls, pointing to ILC responsiveness to the altered environment (285). The IL-2 rich ESRD plasma promotes STAT5 phosphorylation of ILC2s leading to expansion and activation (285). An inverse correlation between circulating ILC2 abundance and infectious complications, as well as elevated IL-33 suggest ILC2 activation as a protective mechanism in ESRD (286). These findings are supported by increased protection from chronic kidney disease by IL-33-induced ILC2 expansion and elevated eosinophil recruitment (287). In contrast, CD56^bright^ NK cells are positively correlated with loss of kidney function in chronic kidney disease and were more abundant in fibrotic biopsies, co-localizing with proximal tubular epithelial cells at sites of tubulointerstitial injury (288). In fibrotic samples, NKp46^+^CD117^+^CD56^bright^ NK cells were the dominant source of IFN-γ and upregulated CD69, implying a role in renal injury and fibrosis (288).

### ILCs in Ischemia-Reperfusion Injury

IRI occurs when temporary disruptions in blood flow cause hypoxic stress and injury to the kidney. Several lines of evidence suggest that ILCs influence IRI severity. Anti-asialo-GM1 and anti-NK1.1 depletion, or NKG2D blockade ameliorated IRI and prevented killing of Rae-1-expressing tubular epithelial cells by NK cells in mice (289, 290). Interactions between co-stimulatory receptor 4-1BB on NK cells and its activating ligand 4-1BBL on epithelial cells activate NK cells and recruit neutrophils *via* epithelial cell-derived CXCL1 and CXCL2 (291). Together, these results support a role for NK cell-epithelial cell interactions in aggravating IRI.

IL-25 and IL-33 administration expands ILC2s in mice, attenuating IRI and promoting recovery post ischemic injury (292, 293). ILC2 depletion using anti-CD90 treated *Rag1^-/-^* mice abolished the protective effect of IL-33 administration (293). Further, adoptive transfer of either murine (into C57BL/6) or human ILC2s (into NSG (NOD.Cg-*Prkdc^scid^ Il2rg^tm1Wjl^*/SzJ) mice) conferred protection from IRI, while AREG-deficient ILC2s failed to abrogate IRI (293). In contrast, Liang et al. reported IL-33 treatment worsened disease scores and fibrosis in mice post-IRI (294). The timing and duration of IL-33 administration and experimental endpoints may account for differences in results, specifically that prolonged IL-33 administration may be detrimental, underscoring the importance of balance between tissue repair and fibrosis processes required to achieve tissue homeostasis.

Despite evidence that expansion or adoptive transfer of ILC2s is beneficial, depletion of ILC2s did not negatively impact IRI, as mice with reduced (*Rora^fl/+^Il7r^cre/+^*), depleted (*Icos^dtr/+^ Cd4^cre/+^*), or deficient for ILC2s (*Rora^fl/fl^Il7r^cre/+^)*, had no effect on IRI, suggesting ILC2 functions can be compensated for by other cell types (295). A regulatory ILC population (Lin^-^ CD127^+^ CD25^+^ IL-10^+^) was identified in mice that limited ILC1s and pro-inflammatory macrophages in an IL-10- and TGF-β-dependent manner (15). Reduced tissue damage was noted in experimental IRI when these cells were expanded in *Rag^-/-^* mice (15). Notably, endogenous ILCregs in IRI produced less IL-10 than expanded ILCregs, suggesting that endogenous ILCreg function is impaired in IRI. While the human counterpart of this regulatory ILC subset was identified, confirmation of their function is needed (15).

### ILCs in Lupus Nephritis

ILCs have been linked to kidney autoimmune pathologies such as lupus nephritis (LN), a manifestation of systemic lupus erythematosus (SLE). Arazi et al. identified two distinct NK cell populations within LN kidney tissue by scRNAseq, annotated as CD56^dim^CD16^+^ NK cells and tissue-resident CD56^bright^CD16^-^ NK cells (296). CD56^+^ NK cells from SLE patients show an impaired antibody-dependent cellular cytotoxicity due to reduced CD3_ζ_ signaling upon NCR engagement (297). Altered abundance of circulating ILCs in LN is associated with disease severity, with increased ILC1s across disease scores and decreased ILC3s in moderate to severe disease scores (298, 299). Further, reduced cytokine production and increased PD-1 expression suggest an exhausted ILC state in active disease (299). Specifically, CD117^+^ ILCs, likely ILC progenitors, were markedly decreased in LN and preferentially differentiated into ILC1s when cultured in LN plasma. Blockade of IL-1R reversed this effect, suggesting IL-1β-mediated regulation of the ILC progenitor pool (299). In a murine model of LN, renal ILC3s were the dominant source of IL-22 and were increased in abundance, while IL-22 deficiency ameliorated disease, supporting a pathogenic role for ILC3s in LN, yet whether an analogous mechanism applies to humans is unknown (300). These studies collectively support dysregulation of ILCs in SLE and LN.

## ILCs in the Female Reproductive System

**Figure 9:**
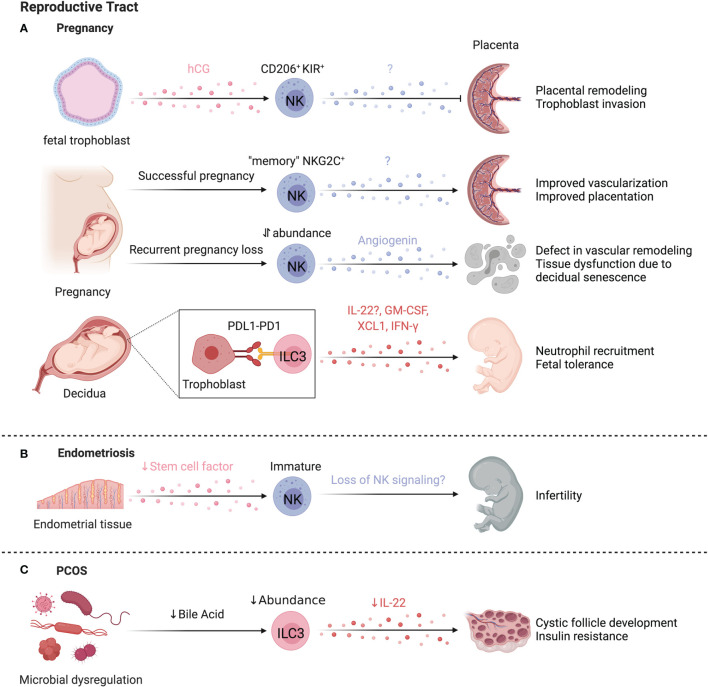
ILCs in the reproductive system. Most ILC data focuses on the female reproductive tract, where NK cells have established roles in pregnancy **(A)**. uNK cells are regulated by human chorionic gonadotropin (hCG) released by fetal trophoblasts, signaling through CD206 (mannose receptor) to facilitate placental remodeling. Women with recurrent pregnancy losses show an increase in uNK cells in the endometrium, coinciding with thickened spiral artery walls and the resulting vascular remodeling affecting the blood flow to the fetus. In support of this, uNK cells from women with recurrent miscarriages also produced more angiogenic factors, fibroblast growth factors, and vascular endothelial growth factors. A special subset of memory uNK cells expressing higher levels of NKG2C contribute to improved reproductive success and lower incidence of pregnancy complications in subsequent pregnancies. In the human decidua, ILC3s express PD-1 and TIM-3, regulating ILC3 cytokine production, in particular IL-33. ILC3-trophoblast interactions may promote fetal tolerance during the first trimester *via* PD-1:PDL-1 interactions, supported by lower PD-L1 levels in trophoblasts of spontaneous abortions compared to healthy terminated pregnancies. **(B)** Reduced stem cell factor leads to a higher proportion of immature uNK cells in endometriosis, potentially contributing to infertility associated with endometriosis. **(C)** In a model of PCOS, microbial dysregulation led to reduced bile acids needed to support ILC3 function. The reduced ILC3 abundance and IL-22 production led to cystic follicle development and insulin resistance. Created with Biorender.org.

ILCs have established roles within the reproductive system and influence pregnancy outcomes (**Figure 9A**). While group 1 and group 3 ILCs are abundant in uterine tissue and participate in the dynamic regulation of reproductive health, little is known about the role of low abundance ILC2s (301).

### ILCs in Pregnancy

Pregnancy is a unique case where non-self is protected from immune-mediated rejection. Dynamic changes occur in both maternal and fetal tissues as pregnancy progresses. The uterine mucosa undergoes cyclic remodeling, termed decidualization, in which the endometrium thickens in preparation for implantation (302). For implantation and placentation to occur, the decidua must be invaded by fetal tissue (trophoblast cells) (303). Together, maternal and fetal tissue interactions promote successful implantation, placentation, and arterial spiralization to facilitate blood and nutrient supply to the developing fetus (303).

Uterine NK (uNK) cells are important cellular regulators of fetal implantation and protect against maternal rejection of the fetus. These uNK cells are the most abundant leukocytes present in the uterine mucosa and exhibit differential functions and limited cytotoxicity compared to cNK cells (304). Despite the discovery of uNK cells in the early 1990’s, the function of uNK cells in healthy and abnormal pregnancy is still the subject of intense research. Uterine NK cells contribute to placental remodeling, striking a balance between excessive trophoblast infiltration and defective placentation, regulated by KIR and MHC-I interactions (305). In general, activating receptor ligation improves reproductive success by promoting trophoblast invasion and vascular transformation (306). Human chorionic gonadotropin (hCG) released by the implanting fetal trophoblast induces uNK cell proliferation through hCG N-linked carbohydrate recognition by CD206 (mannose receptor) on uNK cells, establishing a pathway of uNK cell regulation by the implanting embryo (307).

ScRNAseq defined three distinct subsets of decidual NK cells (dNK cells) in humans. All subsets expressed *CD49A* and *CD9*, dNK1 cells expressed *CD39*, *CYP26A1* and *B4GALNT1*, dNK2 cells expressed *ANXA1* and *ITGB2*, and dNK3 cells expressed *ITGB2*, *CD160*, *KLRB1* and *CD103* (308). In particular, the highly granular and metabolically active dNK1 cells are hypothesized to interact with extravillous trophoblast cells because of high-level expression of KIRs and other HLA-molecule receptors (308). Unlike dNK2 and dNK3 cells, dNK1 cells were mostly IFN-γ^-^ in response to stimulation (309). Computational predictions suggest the mechanism behind the prevention of an inflammatory immune response relies on immune-tissue crosstalk (308). Decidual stromal cells highly express *LGALS9* and *CLEC2D*, pointing to potential NK cell inhibition *via* interaction with TIM-3 and KLRB1 (308). In line with the scRNAseq findings, Huhn et al. confirmed three subsets of dNK cells by mass cytometry with differential expression of the transcription factors TBET and EOMES (309). NK cells in the uterus or decidua acquire KIRs and CD39 along a developmental trajectory, corresponding to increased immunomodulatory and angiogenic function (310). With KIR acquisition, dNK cell expression of LILRB1, Ki-67, NKp30, and Granzyme B increased while NKG2D, CD161, and TBET decreased, unlike the relatively stable expression of these markers on cNK cells (309). Once acquired, the expression of KIRs remain remarkably stable for successive menstruation cycles (311). In another departure from cNK cells, granules were ~3 times larger in dNK cells and as KIR expression increased, degranulation and cytokine production decreased, supporting that dNK cells are phenotypically and functionally unique (309).

High NKG2C marks uNK cells that have acquired memory, or “trained immunity”, contributing to improved reproductive success in subsequent pregnancies by improving vascularization and placentation (312, 313). Greater abundance of uNK cells and aberrant function resulting in higher expression of angiogenic factors in the endometrium coincides with thickening of the spiral artery walls, suggesting alteration of uNK-mediated vascular remodelling as cause for recurring miscarriage (314, 315). However, lower abundance of uNK cells is also associated with recurrent pregnancy losses through reduced decidual-uNK cell interactions. Chronic senescence of decidual cells leads to tissue dysfunction not conducive to successful pregnancies and uNK cells selectively eliminate senescent decidual cells *via* NKG2D, while differentiating decidual cells support and recruit uNK cells with *CXCL14*, *IL-15* and *TIMP3* (316, 317). Indeed, endometrial biopsies from patients with recurrent pregnancy loss exhibit excessive decidual senescence and reduced uNK cell abundance, indicating that a balanced co-operation between decidual cells and uNK cells promotes healthy pregnancies (316).

dNK cells also protect against pregnancy loss from trophoblast bacterial infection. Here, rather than forming a cytotoxic synapse, dNK cells transfer granulysin to infected trophoblasts using nanotube connections, killing the intracellular bacteria without killing the trophoblast (318). This pathway could account for NK-mediated host defense in a setting that aims to avoid excessive tissue damage.

Other ILCs also have defined roles in pregnancy and reproductive conditions. Vacca et al. reported ILC3s in human decidua express PD-1 and TIM-3, which regulate ILC3 cytokine production, most notably IL-22 (319). Since trophoblast cells are PD-L1^high^, ILC3-trophoblast interactions may promote fetal tolerance during the first trimester (319). In support of this, PD-L1 levels were much lower or nonexistent in the trophoblast cells of spontaneous abortions compared to healthy terminated pregnancies (319). Subsets of CD127^hi^CD117^hi^AhR^hi^CD94^-^CD56^+/-^NKp44^+/-^ decidual ILC3s were capable of producing GM-CSF, XCL1 and low levels of IFN-γ upon stimulation (309). NCR^+^ILC3s correlate with neutrophil abundance in the human decidua and produce GM-CSF and CXCL8, supporting neutrophil recruitment and survival (320). Based on lower decidual neutrophil numbers in patients with miscarriages, Croxatto et al. hypothesize that the ILC3-neutrophil axis is beneficial, particularly in early stages of pregnancy (320).

### ILCs in Endometriosis

Pathological functions of uNK cells are linked to endometriosis, a condition affecting ~10% of women where endometrial tissue grows outside of the uterus, resulting in debilitating pain and infertility. Patients with endometriosis have increased immature uNK cell counts and lower levels of stem cell factor (SCF) in the endometrial tissue, associated with infertility (**Figure 9B**) (321). Supplementing cultures of immature uNK cells with SCF supports uNK cell maturation (321). Endometrial stromal cells express high levels of SCF, suggesting stromal-uNK cell interactions influence fertility outcomes in endometriosis. SCF receptor expression is also found on helper ILCs, however, their role in endometriosis requires further investigation (322–324). While many questions remain, these reports support a critical role for uNK cell homeostasis in fertility.

Notably, IL-33 elevation is implicated in endometriosis and exogenous IL-33 exacerbates lesion severity and fibrosis dependent on ILC2s in a murine endometriosis model (325). Yet, in endometrial tissue of patients with endometriosis, ILC2s and ILC3s are reduced in abundance relative to non-endometriosis controls (326). Further work will be needed to clarify the role of hILCs in endometriosis.

### ILCs in Polycystic Ovary Syndrome

Separate from protective roles in pregnancy, reduced ILC3 activity is associated with polycystic ovary syndrome (PCOS). PCOS encompasses mixed metabolic and reproductive pathologies, such as irregular ovulation, infertility, hyperandrogenism, insulin resistance, and adipose tissue inflammation associated with a complex etiology including hormonal dysregulation and heritability (327). Reductions in bile acids due to microbial dysregulation decreased IL-22 production by ILC3s and promoted insulin resistance and cystic follicle development in a murine PCOS model (328). This was reversed by supplementing missing bile acids or IL-22, supporting a link between the gut and fertility (328). However, hormonal imbalances impact ILC function in PCOS. For example, progesterone driven IL-15, IL-18 and CXCL10 expression promote uNK cell recruitment and proliferation, and are altered in PCOS endometrial tissue (**Figure 9C**) (329). These findings indicate that tissue-ILC and microbiota-ILC interactions critically regulate reproductive homeostasis on multiple levels.

## ILCs in the Heart

**Figure 10:**
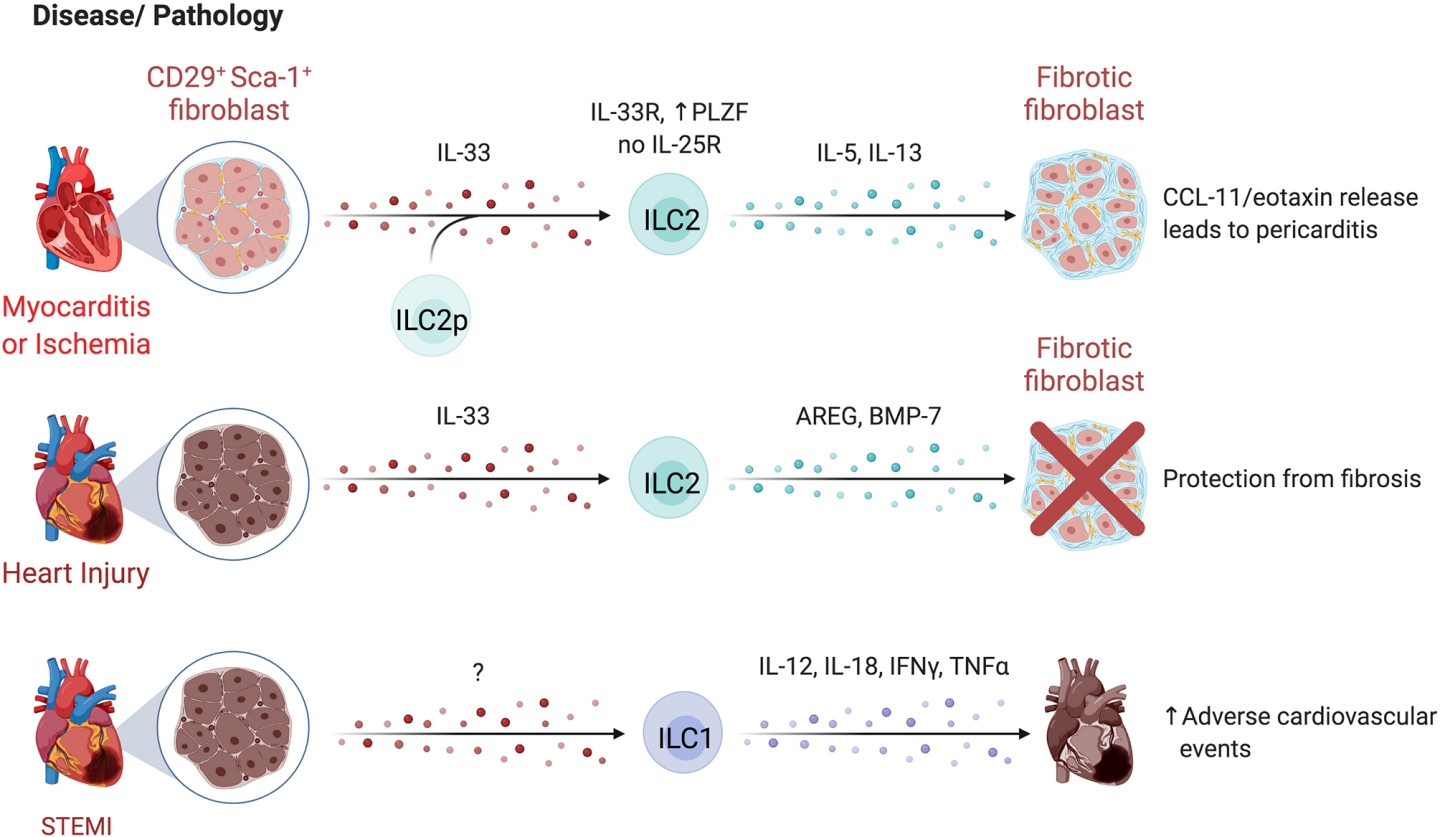
ILCs in the heart. Limited studies have examined ILCs in the heart. In mice, IL-33 administration or CD27^+^Sca-1^+^ fibroblast-derived IL-33 expands ILC2s in the pericardium, driving cardiac fibroblasts to secrete CCL-11/eotaxin, leading to the accumulation of eosinophils. In humans, CD127^+^ ILCs increase in the pericardial fluid during in cardiac disease, particularly during pericarditis. Separately, the expansion of ILC2s by IL-33 was cardioprotective after injury, reducing fibrosis. Within 12 hours of acute ST-segment elevation myocardial infraction (STEMI), ILC1 elevation peaks. Higher ILC1-associated IL-12, IL-18, IFN-γ, and TNF-α levels lead to a higher risk of major adverse cardiovascular events. Created with Biorender.org.

In mice, NK cells account for ~3% of cardiac immune cells, ILC2s for ~1.7%, and ILC1s for 0.2%, while ILC3 abundance is negligible (330). Compared to lung ILC2s, murine cardiac ILC2s had lower expression of ICOS, CD25, and Ki-67, and higher expression of Sca-1 and GATA3 (330). Only 2% of cardiac ILC2s in mouse were donor-derived after 2 months of parabiosis, indicating that ILC2s are a stable tissue-resident population in the heart (330). Cardiac-resident ILC2s respond to IL-33 but not IL-25, and a committed cardiac ILC2 precursor (ILC2p) in mice and humans exists in a quiescent state with the capability to differentiate into ILC2s in response to myocardial infarct or myocarditis (331). The existence of undifferentiated ILC2p within tissues has been observed before and suggests a role for this pool of precursors as a reservoir for ILCs to protect from tissue damage (332). Cardiac ILC activity has been implicated in several disease models (**Figure 10**).

### ILCs in Atherosclerosis and Coronary Artery Disease

Conflicting findings on ILC subset functions in atherosclerosis and coronary artery disease (CAD) have been reported. Selathurai et al. found murine NK cells were detrimental to atherosclerotic disease, increasing lesion size in a Perforin- and Granzyme B-dependent manner, while Nour-Eldine et al. found no effect of NK cells on lesion development using a distinct genetic depletion model, likely accounting for divergent findings (333, 334).

In humans, a study of acute ST-segment elevation myocardial infarction (STEMI) found elevated circulating ILC1s within 12 hours of symptom onset which produced more IL-12, IL-18, IFN-γ, and TNF-α, and were associated with a higher risk of major adverse cardiovascular events (335). In contrast, NK cells were reduced and have lower cytotoxicity in CAD patients, both in the case of stable angina and incidence of myocardial infarction or unstable disease (336–338). During follow-up, patients who failed to reconstitute their peripheral NK cells post myocardial infarction had higher levels of serum IL-6 and exhibited characteristics of metabolic syndrome, suggesting poor NK cell recovery corresponds with low-grade inflammation (336). Recovery of NK cells in CAD patients is potentially self-regulated, as apoptotic NK cells both respond to and produce FasL, which is elevated in serum and correlates with NK cell levels and apoptotic susceptibility (339). Increased proportions of CD56^bright^ NK cells were identified in carotid plaques compared to autologous peripheral blood, and greater NK cell infiltration corresponded with symptomatic versus asymptomatic CAD patients (340). Soluble B7-H6 levels of 250 pg/ml were detected in symptomatic patients but not in asymptomatic patients or healthy controls (340). Notably, B7-H6 can interact with NKp30, yet further studies are needed to directly assess B7-H6 and NK cell interactions in this context. Circulating NK cells from atherosclerotic patients had higher TIM-3 expression than healthy controls, with the greatest levels in those with unstable plaques (341). TIM-3 blockade reduced the death of NK cells cultured in TNF-α, suggesting that TIM-3 promotes cytokine-induced NK cell apoptosis in atherosclerosis (341). Whether NK cells are preferentially recruited to unstable carotid plaques, or functionally contribute to plaque destabilization requires additional study (340).

ILC2s appear to have cardioprotective functions based on mouse models. ILC2s protect from cardiac fibrosis and are enhanced by exogenous IL-33, producing AREG and BMP-7 to support cardioprotective responses to injury (342). Expansion of ILC2s reduces atherosclerosis severity and lesion size, while genetic ablation (*Staggerer/Rora^Flox^-CD127^Cre^*) exacerbates disease (343, 344). Notably, protection is IL-5 and IL-13-dependent, recruiting eosinophils and polarizing macrophages towards an anti-inflammatory phenotype (342, 343). Further, pericardial and cardiac ILC2s expand early post-experimental myocardial infarction, peaking at day 3 before returning to homeostatic levels, while the absence of ILC2s impairs cardiac remodeling and results in larger areas of scarring (345). Together, this supports a role for cardiac-resident ILC2s in directing repair pathways in response to injury.

### ILCs in Cardiac Inflammation

ILC expansion has been observed in patients with pericarditis (346). In contrast to cardioprotective findings above, ILC2s have been implicated in pericarditis pathology. Exogenous IL-33 expanded murine pericardial ILC2s, driving cardiac fibroblasts to secrete CCL11/eotaxin-1 and recruit eosinophils, initiating pericarditis (346). Pericardial fluid from humans revealed an elevated frequency of CD127^+^ ILCs in patients with cardiac disease versus controls, indicating that ILCs are also involved in human pericardial pathology (346). In an opposing role, NK cell depletion led to greater inflammation and fibrosis of the heart, dependent on NK cell-mediated prevention of eosinophilic infiltration (347). A possible cross-regulation of NK cells and ILC2s during cardiac inflammation should be investigated.

Sex differences in mortality and morbidity of Coxsackievirus B3 (CVB3) viral myocarditis may also reflect sex-based regulation of NK cell function. Male mice experience greater morbidity and mortality from myocarditis following CVB3 infection, with increased IFN-γ^+^ NK cell infiltration in cardiac tissue (348). Ovariectomized or sexually immature female mice show similar susceptibility to infection-triggered myocarditis when compared to male mice, while estrogen-treated male mice had ameliorated myocarditis (348). CVB3-stimulated NK cells cultured with estrogen down-regulated T-bet expression and consequently had reduced IFN-γ production, indicating that regulation of T-bet expression by estrogen might underlie the decreased IFN-γ^+^ NK cell infiltration in female mice and contribute to sex differences in myocarditis, in line with prior reports of hormonal regulation of other ILC subsets (100, 348).

## Adipose ILCs

Adipocytes are critical regulators of energy and glucose homeostasis. They are a heterogeneous population of cells, comprising energy-storing white adipocytes, thermogenic brown adipocytes that express uncoupling protein-1 (UCP-1) to dissipate energy as heat, as well as beige adipocytes that reside within white adipose tissue (WAT) and upregulate UCP-1 in response to environmental cues (349). Beige adipocyte accumulation protects from insulin resistance, and regulation of the beiging process has become an attractive therapeutic target for metabolic dysregulation and type 2 diabetes (350).

**Figure 11:**
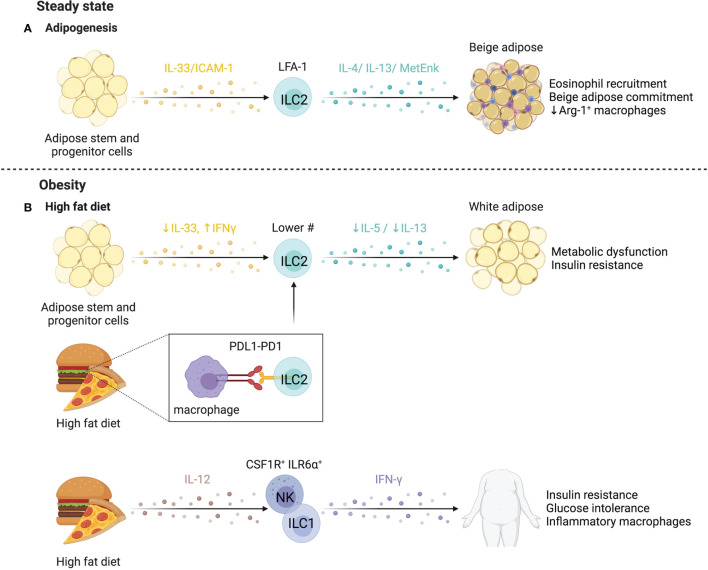
ILCs in adipose tissue. **(A)** Using mostly mouse models, ILC2s are linked to adipogenesis, where they maintain homeostasis and prevent obesity by promoting adipose beiging. ILC2s are sustained by IL-33 from adipose stem and progenitor cells (ASPCs) and required stromal cell interactions *via* ICAM-1 and LFA-1. The resulting release of IL-4, IL-13 by ILC2s promotes eosinophil recruitment *via* stromal cell-derived eotaxin (CCL11). IL-33 stimulates ILC2-produced methionine-enkephalin (MetEnk), an endogenous opioid-like peptide that promotes adipose beiging. **(B)** Both obese humans and mice, have reduced ILC2s in the white adipose tissue due to diet-driven impairment of IL-33 production by ASPCs. The infiltration of IFN-γ-producing ILCs actively represses cytokine release by ILC2s and propagates ILC2 inhibition through *via* PD-1 and macrophage-expressed PD-L1. A unique subpopulation of CSF1R^+^IL16Rα^+^ NK cells and increased ILC1 abundance positively correlates with glucose intolerance and insulin resistance. Overall, reduction of adipose ILC2s fosters metabolic dysfunction, insulin resistance and obesity. Created with Biorender.org.

ILC2s are the dominant hILC subtype identified in adipose tissue, with phenotypic variation across different murine adipose compartments: ILC2s in para-aortic adipose tissue have an inflammatory phenotype defined by IL-25 responsiveness and high KLRG1 expression, whereas peri-gonadal adipose ILC2s are IL-33 responsive, expressing ST2 (343, 351). Intriguingly, mouse ILC2s highly express bone morphogenetic protein (*Bmp)2* and *Bmp7*, which promote adipocyte differentiation, while ILC-deficient mice have more CD34^+^PDGFRα^+^ precursor adipocytes, supporting a role for ILCs in adipogenesis (352). Under homeostatic conditions, ILC2s orchestrate immune responses in adipose tissues by recruiting eosinophils, promoting alternative activation of macrophages, and regulating beiging, glucose catabolism, and insulin sensitivity in adipose tissues (**Figure 11A**) (353, 354). In support of this, depletion of IL-5 and IL-13-producing cells (mainly ILC2s) corresponded to a reduction of eosinophils and Arg-1^+^ adipose macrophages in visceral adipose tissue (355). ILC2s also promote Treg responses through ICOSL and OX40L co-stimulation in adipose tissues, critical for supporting insulin sensitivity (356–358).

Mouse studies support that ILC2s can directly promote adipose beiging, supporting homeostasis and preventing obesity (5, 359, 360). ILC2s are sustained by IL-33 from adipose stem and progenitor cells (ASPCs) (360). Stromal ICAM-1 interactions with LFA-1 on ILC2s promotes ILC2 activation and proliferation in adipose tissue, while IL-4/IL-13 expression by ILC2s induces eotaxin (CCL11) expression in stromal cells, supporting eosinophil recruitment (361). Peritoneal IL-33 administration expands adipocyte precursors and promotes beige lineage commitment in an ILC2-dependent manner, as effects are abolished in the absence of ILC2s and when adipocyte precursors are not receptive to IL-4/IL-13 signaling (*Il4ra^fl/fl^Pdgfra^Cre^*) (359). An alternative mechanism of ILC2-dependent beiging of mouse adipose tissue was proposed by Brestoff et al. who found IL-33-stimulated ILC2s produce methionine-enkephalin (MetEnk), an endogenous opioid-like peptide which induces WAT beiging (5). Overall, these studies demonstrate that ILC2-dependent eosinophil-derived IL-4 and ILC2-derived IL-13 and/or MetEnk directly promote murine adipocyte precursor proliferation and beige lineage commitment (5, 359).

Group 1 ILCs are largely resident in murine adipose tissue (362). Although their homeostatic role is poorly understood, ILC1s regulate adipose macrophage homeostasis (363). Alternatively activated macrophages scavenge potentially cytotoxic molecules released during adipose tissue remodeling and upregulate stress ligands (i.e. Rae-1) at steady state, and their selective depletion by adipose type 1 ILCs prevents stress-induced inflammation in macrophages during homeostatic tissue remodeling (363).

### ILCs in Obesity

Dysregulation of the immune environment associated with obesity can lead to metabolic dysfunction and insulin resistance, driving type 2 diabetes (T2D) (364). ILC dysregulation has been implicated in obesity (**Figure 11B**). In obese humans and mice, ILC2s are reduced in WAT, possibly due to reduced IL-33 production by ASPCs in response to high fat diet (5, 360, 365). High PD-1 expression on ILC2s reduced IL-5 and IL-13 production, an effect partially rescued by macrophage depletion, suggesting PD-1/PD-L1 interactions between ILC2s and macrophages dampens ILC2 function in obese conditions in mice (365). Additionally, adipocyte-derived soluble ST2 is induced by obesity and interrupts IL-33 signaling, impairing ILC2 homeostasis (366). Infiltration of IFN-γ-producing cells also contributes to reduced ILC2 abundance and function, as IFN-γ directly represses ILC2s and counteracts IL-33 (357).

Obese mice fed a high fat diet had adipose tissue-specific IL-12-dependent accumulation of ILC1s with elevated IFN-γ production, resulting in insulin resistance and glucose intolerance (362, 367). Interestingly, CD56^dim^ CD16^–^ ILCs accumulating during obesity have reduced cytotoxicity, a potential secondary mechanism contributing to macrophage accumulation and glucose intolerance (363). Wensveen et al. demonstrated NKp46 on adipose-resident mouse NK cells may regulate this effect (368). High fat diet-induced obesity triggered the expression of NCR1 ligands on adipocytes which promoted local NK cell proliferation and production of IFN-γ, inducing the differentiation of pro-inflammatory macrophages and promoting insulin resistance (368). Wang et al. further found that the ILC1 IFN-γ-dependent expansion of pro-inflammatory macrophages exacerbated adipose fibrosis by promoting TGF-β1 and pro-fibrotic programs in macrophages, resulting in higher collagen deposition (367).

In agreement with murine models, circulating and adipose ILC1s are increased in obese patients, especially those with T2D, and the abundance of ILC1s positively correlates with measures of glucose intolerance and insulin resistance (367). A unique subpopulation of CSF1R^+^IL6Rα^+^ NK cells is expanded in human and murine obesity (369). Selective depletion of this subset (*Csf1r^-loxSTOPlox-DTR^ x Ncr^Cre^*) resulted in decreased weight gain, better glucose tolerance, and insulin responsiveness in mice fed a high fat diet (369). Further, the expression of RORγt, lymphotoxin and IL-22 all elevated weight gain and adipose tissue size, paralleling findings that IL-22 from Th17 cells exacerbates inflammation in obesity (370, 371). The regulation of metabolism by intestinal ILC3s suggests a gut-adipose axis that remains to be explored. Overall, ILC2s mediate adipose homeostasis and are dysregulated in obesity, while ILC1s and potentially ILC3s have a role in exacerbating inflammation.

## Concluding Remarks

Multiple parallels and differences between murine and human ILCs exist. Their evolutionarily conserved transcriptional programs and functional similarity emphasizes their importance across multiple distinct phylogenetic branches. However, a better understanding of homologies and analogies in their surface receptor expression and function are needed to inform conserved mechanisms underlying responses to infections, inflammation and malignancies. This will be of particular interest to enhance our understanding NK cell biology, where receptors regulating NK cells responses differ between mouse and human, but different receptors often perform similar function.

The distinct living conditions of mice and humans require conserved and specified adaptations of organs and tissues to environmental triggers. ILCs as regulators of tissue homeostasis adapt to these species-specific environments. Shared and differing microbiota within humans and mice may explain conserved and distinct functions of ILCs across these organisms. Gnotobiotic technologies, humanized mice, knock-out mouse models or adoptive transfer experiments are suitable to investigate these differences but bring their own pitfalls. Beyond these challenges, a key obstacle for the study of ILCs *in situ* is the scarcity of ILC-specific models available to tease out cell-specific or even organ-specific functions. Adding to this, ILCs typically function in cellular networks to influence the outcome of a given immune response. To properly understand their function and tease out any redundancy, more systems-based approaches are needed, particularly in humans (295, 372, 373).

Other roadblocks in understanding the role of various ILCs in homeostasis and disease are studies designed only to link the presence or absence of ILCs with disease outcome. This is especially evident in reports of NK cell function. Reporting expansion or reduction of NK cells as having a protective or detrimental effect assumes a homogeneous function of NK cells. While classically, NK cell function has been cytotoxic and inflammatory, NK cells have also been cast in an immunoregulatory role where they dampen an immune response (19). Additionally, identifying NK cells as CD3^-^CD56^+^ does not rule out other non-cytotoxic ILC1s and ILC3s that can share CD56 expression, and does not address the large degree of heterogeneity within NK cells (374). Untangling the identity and functional capacity of distinct CD3^-^ CD56^+^ populations may help to clarify contradictory findings.

The plasticity of ILCs makes definitively assigning them as “good” or “bad” quite problematic. Sometimes ILCs with identical or similar surface phenotype may be functionally distinct (206). While this may be context dependent, ILC plasticity may be partially responsible for conflicting reports of their function in disease. Future studies should consider assessing the functional role of ILC subsets correlated with ILC transcriptional and epigenetic profiles to identify mechanisms underlying distinct ILC functions and whether some level of ‘trained’ immunity contributes to differing findings. Additionally, it remains unclear if the tissue-specific functions of ILCs are due in part to the existence of specific subsets of ILCs that home to their niche, or instead these different functions are a directly due to microenvironment signals leading to niche adaptation. It is also entirely possible that both cases are true and contribute to establishing tissue-specific ILC functions. Moving forward, the characterization of tissue-specific networks and niches for ILCs will transform our understanding of ILC functions and underlying mechanisms controlling their tissue adaptations.

## Author Contributions

All authors contributed to conceptualization, topic curation, writing and editing of the manuscript. Figures were designed by LN, with input from JM, AM, and SC. All authors contributed to the article and approved the submitted version.

## Funding

JMM was supported by Queen Elizabeth II, Dick and Peggy Sharpe, and Peterborough KM Hunter Scholarships. LN was supported by an Ontario Graduate Scholarship and a NSERC-PGS award. AM's research program is supported by Canadian Institutes for Health Research (CIHR, 388337) and Natural Sciences and Engineering Research Council of Canada (NSERC, RGPIN-2019-04521), and is a Tier 2 Canadian Research Chair in Mucosal Immunology. SQC's research program is supported by funding from CIHR (168960 and 169084) and a NSERC (RGPIN-2021-03672), and is a Medicine By Design Investigator (Canada First Research Excellence Fund).

## Conflict of Interest

The authors declare that the research was conducted in the absence of any commercial or financial relationships that could be construed as a potential conflict of interest.

## Publisher’s Note

All claims expressed in this article are solely those of the authors and do not necessarily represent those of their affiliated organizations, or those of the publisher, the editors and the reviewers. Any product that may be evaluated in this article, or claim that may be made by its manufacturer, is not guaranteed or endorsed by the publisher.

## Glossary

**Table:**

AD	Atopic dermatitis
Ahr	Aryl hydrocarbon receptor
APC	Antigen presenting cell
AREG	Amphiregulin
ASPCs	Adipose stem and progenitor cells
CAD	Coronary artery disease
CD	Crohn’s disease
ChAT	Choline acetyltransferase
COPD	Chronic obstructive pulmonary disease
cNK cells	Conventional NK cells
CNS	Central nervous system
CP	Cryptopatches
CVB3	Coxsackievirus B3
DC	Dendritic cell
dNK cells	Decidual Natural Killer cells
EAE	Experimental autoimmune encephalitis
EOMES	Eomesodermin
ESRD	End stage renal disease
FasL	Fas ligand
Ffar2	Free Fatty Acid Receptor 2
FHL2	Four And A Half LIM Domains 2
GITR	Glucocorticoid-induced TNFR-related protein
GPR34	G Protein-Coupled Receptor 34
HBV	Hepatitis B virus
hCG	Human chorionic gonadotropin
HCV	Hepatitis C virus
HDM	House dust mite
hILC	helper ILC
IBD	Inflammatory bowel disease
ID	Inhibitor of DNA binding
IFN-γ	Interferon gamma
IL	Interleukin
ILC	Innate lymphoid cell
ILC1	Group 1 innate lymphoid cell
ILC2	Group 2 innate lymphoid cell
ILC2_10_	IL-10 producing ILC2
ILC3	Group 3 innate lymphoid cell
ILCreg	Regulatory ILC
ILF	Isolated lymphoid follicle
IRI	Ischemia reperfusion injury
KIR	Killer immunoglobulin receptor
KLRG1	Killer cell lectin like receptor G1
LN	Lupus nephritis
LP	Lamina propria
LTi	Lymphoid tissue inducer
LXA4	Lipoxin A4
MHC-I	Major histocompatibility complex class I
MHC-II	Major histocompatibility complex class II
miRNA	MicroRNA
MS	Multiple Sclerosis
NAFLD	Non-alcoholic fatty liver disease
NASH	Non-alcoholic steatohepatitis
NCR	Natural cytotoxicity receptor
PCOS	Polycystic ovary syndrome
RA	Retinoic acid
RORA	RAR-related orphan receptor A
RORC	RAR-related orphan receptor C
RSV	Respiratory syncytial virus
SCF	Stem cell factor
SCFA	Short chain fatty acid
scRNAseq	Single cell RNA sequencing
SLE	Systemic lupus erythematosus
sLT	Surface lymphotoxin
SPM	Specialized pro-resolving mediator
T2D	Type 2 diabetes
TBET	T-box transcription factor
Tfh	T follicular helper
TIGIT	T cell immunoreceptor with Ig and ITIM domains
TNF	Tumor necrosis factor
Treg	Regulatory T cell
TSLP	Thymic stromal lymphopoietin
UCP-1	Uncoupling protein 1
uNK cells	Uterine Natural Killer cells
VIP	Vasoactive intestinal peptide
WAT	White adipose tissue
AD	Atopic dermatitis
Ahr	Aryl hydrocarbon receptor
APC	Antigen presenting cell
AREG	Amphiregulin
ASPCs	Adipose stem and progenitor cells
CAD	Coronary artery disease
CD	Crohn’s disease
ChAT	Choline acetyltransferase
COPD	Chronic obstructive pulmonary disease
cNK cells	Conventional NK cells
CNS	Central nervous system
CP	Cryptopatches
CVB3	Coxsackievirus B3
DC	Dendritic cell
dNK cells	Decidual Natural Killer cells
EAE	Experimental autoimmune encephalitis
EOMES	Eomesodermin
ESRD	End stage renal disease
FasL	Fas ligand
Ffar2	Free Fatty Acid Receptor 2
FHL2	Four And A Half LIM Domains 2
GITR	Glucocorticoid-induced TNFR-related protein
GPR34	G Protein-Coupled Receptor 34
HBV	Hepatitis B virus
hCG	Human chorionic gonadotropin
HCV	Hepatitis C virus
HDM	House dust mite
hILC	helper ILC
IBD	Inflammatory bowel disease
ID	Inhibitor of DNA binding
IFN-γ	Interferon gamma
IL	Interleukin
ILC	Innate lymphoid cell
ILC1	Group 1 innate lymphoid cell
ILC2	Group 2 innate lymphoid cell
ILC2_10_	IL-10 producing ILC2
ILC3	Group 3 innate lymphoid cell
ILCreg	Regulatory ILC
ILF	Isolated lymphoid follicle
IRI	Ischemia reperfusion injury
KIR	Killer immunoglobulin receptor
KLRG1	Killer cell lectin like receptor G1
LN	Lupus nephritis
LP	Lamina propria
LTi	Lymphoid tissue inducer
LXA4	Lipoxin A4
MHC-I	Major histocompatibility complex class I
MHC-II	Major histocompatibility complex class II
miRNA	MicroRNA
MS	Multiple Sclerosis
NAFLD	Non-alcoholic fatty liver disease
NASH	Non-alcoholic steatohepatitis
NCR	Natural cytotoxicity receptor
PCOS	Polycystic ovary syndrome
RA	Retinoic acid
RORA	RAR-related orphan receptor A
RORC	RAR-related orphan receptor C
RSV	Respiratory syncytial virus
SCF	Stem cell factor
SCFA	Short chain fatty acid
scRNAseq	Single cell RNA sequencing
SLE	Systemic lupus erythematosus
sLT	Surface lymphotoxin
SPM	Specialized pro-resolving mediator
T2D	Type 2 diabetes
TBET	T-box transcription factor
Tfh	T follicular helper
TIGIT	T cell immunoreceptor with Ig and ITIM domains
TNF	Tumor necrosis factor
Treg	Regulatory T cell
TSLP	Thymic stromal lymphopoietin
UCP-1	Uncoupling protein 1
uNK cells	Uterine Natural Killer cells
VIP	Vasoactive intestinal peptide
WAT	White adipose tissue
